# Redox regulation of K_V_7 channels through EF3 hand of calmodulin

**DOI:** 10.7554/eLife.81961

**Published:** 2023-02-20

**Authors:** Eider Nuñez, Frederick Jones, Arantza Muguruza-Montero, Janire Urrutia, Alejandra Aguado, Covadonga Malo, Ganeko Bernardo-Seisdedos, Carmen Domene, Oscar Millet, Nikita Gamper, Alvaro Villarroel

**Affiliations:** 1 Instituto Biofisika, CSIC-UPV/EHU Leioa Spain; 2 School of Biomedical Sciences, Faculty of Biological Sciences, University of Leeds Leeds United Kingdom; 3 Atlas Molecular Pharma S.L Derio Spain; 4 Department of Chemistry, University of Bath Bath United Kingdom; 5 Department of Chemistry, University of Oxford Oxford United Kingdom; 6 Protein Stability and Inherited Disease Laboratory, CIC bioGUNE Derio Spain; Columbia University United States; The University of Texas at Austin United States

**Keywords:** KCNQ, calmodulin, redox, calcium, EF-hand, signal transduction, *E. coli*

## Abstract

Neuronal K_V_7 channels, important regulators of cell excitability, are among the most sensitive proteins to reactive oxygen species. The S2S3 linker of the voltage sensor was reported as a site-mediating redox modulation of the channels. Recent structural insights reveal potential interactions between this linker and the Ca^2+^-binding loop of the third EF-hand of calmodulin (CaM), which embraces an antiparallel fork formed by the C-terminal helices A and B, constituting the calcium responsive domain (CRD). We found that precluding Ca^2+^ binding to the EF3 hand, but not to EF1, EF2, or EF4 hands, abolishes oxidation-induced enhancement of K_V_7.4 currents. Monitoring FRET (Fluorescence Resonance Energy Transfer) between helices A and B using purified CRDs tagged with fluorescent proteins, we observed that S2S3 peptides cause a reversal of the signal in the presence of Ca^2+^ but have no effect in the absence of this cation or if the peptide is oxidized. The capacity of loading EF3 with Ca^2+^ is essential for this reversal of the FRET signal, whereas the consequences of obliterating Ca^2+^ binding to EF1, EF2, or EF4 are negligible. Furthermore, we show that EF3 is critical for translating Ca^2+^ signals to reorient the AB fork. Our data are consistent with the proposal that oxidation of cysteine residues in the S2S3 loop relieves K_V_7 channels from a constitutive inhibition imposed by interactions between the EF3 hand of CaM which is crucial for this signaling.

## Introduction

The generation of abnormally high levels of reactive oxygen species (ROS) is linked to cellular dysfunction, including neuronal toxicity and neurodegeneration (Abdullaeva et al., 2022; Sahoo et al., 2014; Sohal and Orr, 2012). In addition, ROS are important mediators of normal cellular functions in multiple intracellular signal transduction pathways (Dantzler et al., 2019; Miki and Funato, 2012; Wani and Murray, 2017; Weidinger and Kozlov, 2015). ROS generation induces oxidative modifications and augmentation of M-currents in neurons, which provides protective effects on oxidative stress-related neurodegeneration (Bierbower et al., 2015; Gamper et al., 2006; Vigil et al., 2020). K_V_7 channels, the substrate of the K_V_7-mediated M-current, are among the most sensitive proteins that respond to ROS production (Gamper et al., 2006; Jones et al., 2021; Sahoo et al., 2014).

Superoxide anion radicals (O_2_•−), hydroxyl radicals (•OH), peroxynitrite (ONOO−), and hydrogen peroxide (H_2_O_2_) are the main ROS produced in cells (Mittal et al., 2014). These molecules display different reactivity, concentration and lifetime, and most probably play different roles in signal transduction and oxidative stress. Oxidation of cysteine thiol side chains mediated by H_2_O_2_ is the most recognized and studied redox reversible post-translational modification. Because of its relative stability and ability to cross the plasma membrane, H_2_O_2_ has been shown to be important in a variety of neurophysiological processes, including neurotransmission, ion channel function, and neuronal activity (Gamper and Ooi, 2015; Kamsler and Segal, 2007; Lee et al., 2015; Patel and Rice, 2012).

Augmentation of the M-current can be induced by an external H_2_O_2_ concentration as low as 5 μM (Gamper et al., 2006) or even in the nM range (Abdullaeva et al., 2022). The M-current flow through channels formed of neuronal K_V_7 subunits (K_V_7.2-K_V_7.5, encoded by KCNQ2-5 genes). These tetrameric channels open at the subthreshold membrane potentials and dampen cellular excitability (Adams, 2016; Soldovieri et al., 2011). K_V_7 channels have a core architecture similar to other voltage-dependent potassium channels (Li et al., 2021a; Li et al., 2021b; ; Sun and MacKinnon, 2017; Sun and MacKinnon, 2020; Xu et al., 2013): they have six helical transmembrane domains (S1–S6) with the voltage sensor formed by S1–S4, followed by a pore domain (S5–S6), which continues into a cytosolic C-terminal region. The C-terminus of K_V_7 channels contains five helical regions: helices A–D and TW helix between hA and hB. The latter region forms the calcium responsive domain (CRD) with helices AB adopting an antiparallel fork disposition (Sachyani et al., 2014). Four C-helices from each subunit come together to form a stem perpendicular to the membrane. This stem continues with an unstructured linker that connects to helix D, which forms a tetrameric coiled-coil structure that confers subunit specificity during subunit assembly (Sachyani et al., 2014).

All K_V_7 channels require the association of calmodulin (CaM) to the CRD to be functional (Gamper and Shapiro, 2003; Wen and Levitan, 2002; Yus-Najera et al., 2002). Helices AB are embraced by CaM forming a compact structure just under the membrane that can move as a rigid body, with a region connecting S6 and helix A acting as a hinge (Li et al., 2021a; Li et al., 2021b; Xu et al., 2013). CaM is the main adaptor protein that confers Ca^2+^ sensitivity to an ample array of eukaryotic proteins and is composed of two highly homologous lobes joined by a flexible linker. In solution, each lobe operates almost independently of the other and contains two similar Ca^2+^-binding EF-hands (Sorensen and Shea, 1998). This distinct signaling mediated by each CaM lobe was revealed early in *Paramecium* when it was discovered that mutations at the N-lobe affected a Ca^2+^ operated Na^+^ conductance, whereas mutations at the C-lobe affected a Ca^2+^ dependent K^+^ conductance (Kung et al., 1992).

A structure of the non-neuronal K_V_7.1 subunit trapped in a non-functional conformation with the voltage-sensor disengaged from the pore suggests that the EF3-hand of CaM may interact with the voltage sensor (Kang et al., 2020; Li et al., 2021b; Sun and MacKinnon, 2017) at a site essential for M-current redox modulation (Abdullaeva et al., 2022; Gamper et al., 2006). This 3D configuration has been assumed to confer a preferential use of EF3 during signaling on K_V_7 channels (Chang et al., 2018; Kang et al., 2020; Tobelaim et al., 2017; Zhuang and Yan, 2020).

Here, we address the role of CaM on redox modulation of K_V_7 channels, finding a critical role of EF3-hand. We show that H_2_O_2_ interrupts crosstalk between the S2S3 linker and the EF3-hand of CaM in a Ca^2+^-dependent manner. We have monitored the role of each EF-hand in the gating process of the CRD, characterized by the opening of the AB fork (Bernardo-Seisdedos et al., 2018). By studying purified signaling components in a well-controlled in vitro setting, we avoided unwanted off-target effects arising from interactions with other domains of the channel; we then tested our structural findings in cellulo. The prevailing view is that there is a high degree of cooperativity between the pair of EF-hands within each lobe, such that each lobe operates as a unit regarding Ca^2+^ binding and signaling and that the 3D arrangement is required for EF-hand specific allosteric signaling of K_V_7 channels (Kang et al., 2020; Zhuang and Yan, 2020). In contrast, we show an additional level of specialization whereby just one EF-hand is critical for Ca^2+^ signaling, and the direction of gating can change upon interaction between S2S3 loop domain and solvent-exposed EF-hands. Thus, preferential signaling through EF3 is an intrinsic property not derived from the 3D arrangement revealed by the available K_V_7 structures. The emergence of this novel mode of CaM modulation promises generalization to complexes with the EF-hands interacting with solvent-exposed regions of the target proteins.

## Results

### CaM plays a critical role in H_2_O_2_-mediated regulation of K_V_7.4 channels

**Figure 1.**
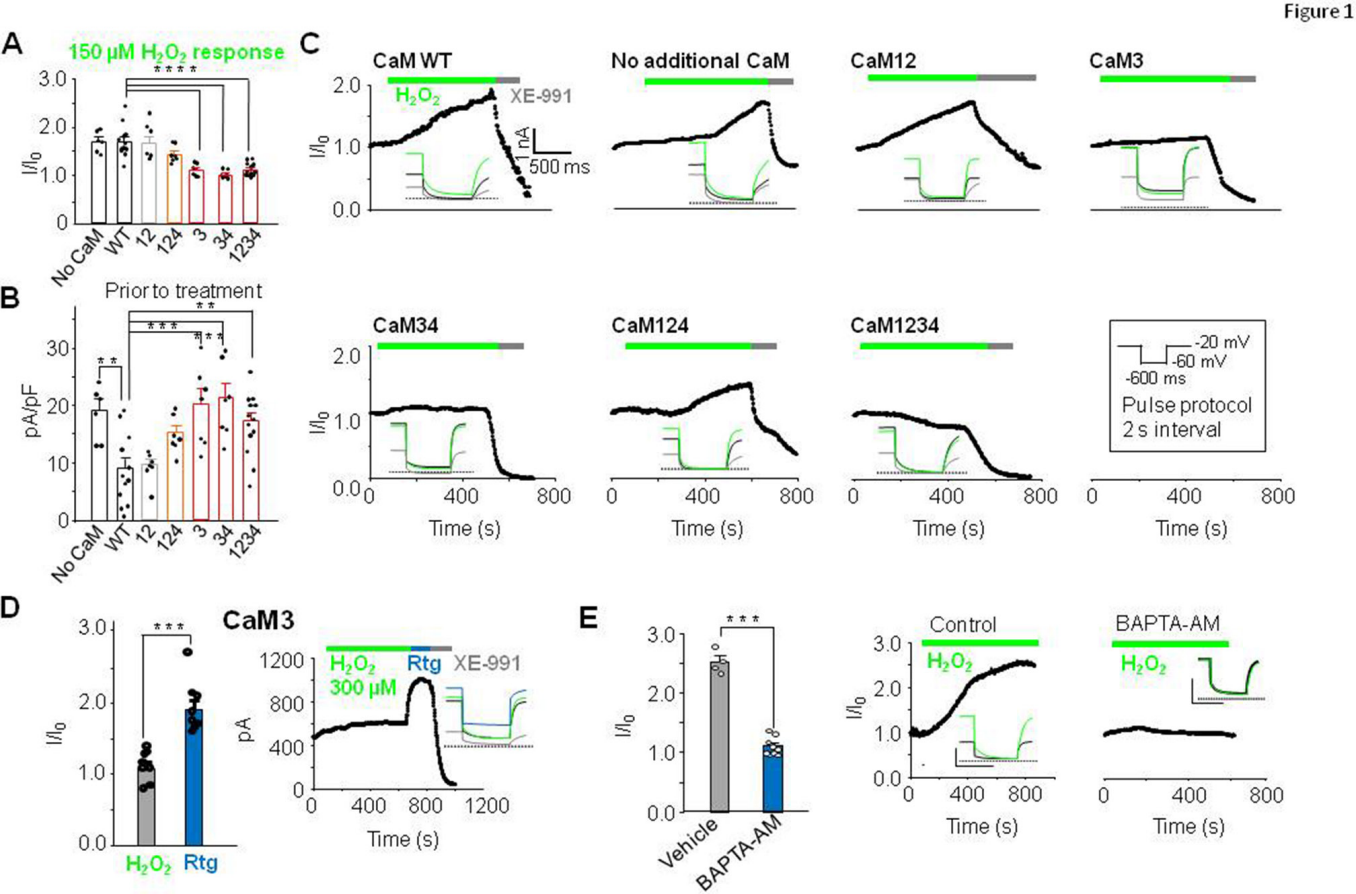
EF3 hand Ca^2+^ binding capacity of CaM is required for H_2_O_2_-mediated potentiation of K_V_7.4. (**A**) Response of K_V_7.4 transfected HEK293 cells to 150 µM H_2_O_2_ (normalized steady-state current at –60 mV, I/I_0_) when transfected with wild-type CaM (CaMWT n=12), mutant CaMs lacking Ca^2+^ binding to one or more EF hands. The number in X-axis of panel B applies and pertains to the EF hand unable to bind Ca^2+^ (CaM12 n=7, CaM124 n=7, CaM3 n=7, CaM34 n=7, and CaM1234 n=13) or with no additional CaM transfected (No CaM, n=6). (**B**) Current density (pA/pF; –60 mV) of K_V_7.4 transfected cells prior to treatment with H_2_O_2_. (**C**) Representative currents at –60 mV in response to 150 µM H_2_O_2_ followed by 10 µM XE-991. Inset: representative current traces from each condition. (**D**) Comparative response of cells transfected with K_V_7.4 and CaM3 to 300 µM H_2_O_2_ and 10 µM retigabine (n=8). (**E**) Ca^2+^ dependence of H_2_O_2_ response in cells transfected with K_V_7.4 and CaMWT. Comparison of 300 µM H_2_O_2_ response in normal or low Ca^2+^ conditions induced by pre-incubation of cells in 10 µM BAPTA-AM for 30 min to chelate intracellular Ca^2+^. Control n=4, BAPTA-AM n=9. Data presented are mean ± SEM, statistical evaluation by independent measures ANOVA with Dunnett’s post hoc, **p<0.01, ***p<0.001, and ****p<0.0001 (A and B). A paired (**D**) or unpaired (**E**) two-tailed T test ***p<0.001 and ****p<0.0001. Figure 1—source data 1.The current voltage relationship of cells transfected with mutant CaM does not differ significantly from CaMWT.

We have previously shown that cysteine residues present in the unusual long linker between S2S3 transmembrane segments of K_V_7 channels are critical for H_2_O_2_-dependent potentiation (Gamper et al., 2006). Recent studies suggest structural and functional interactions between this loop and CaM (Chang et al., 2018; Kang et al., 2020; Sun and MacKinnon, 2017; Tobelaim et al., 2017; Zhuang and Yan, 2020). To test a possible role of CaM in redox modulation, we used the perforated patch clamp method to measure K_V_7.4 activity in response to H_2_O_2_. Human KCNQ4 cDNA was co-expressed in HEK293 cells with either CaM or CaM mutants that, by replacing the aspartate residue with alanine in the first position of the EF-hands, the Ca^2+^ binding ability of the N-lobe (CaM12), the C-lobe (CaM34), or both (CaM1234; Geiser et al., 1991; Keen et al., 1999) is disabled (Figure 1). Cells were held at –20 mV, and 600 ms voltage pulses to –60 mV were applied every 2 s; K_V_7.4 activity was monitored as the outward steady-state current amplitude at –60 mV (Figure 1C). Bath-application of 150 μM H_2_O_2_ induced a clear augmentation of steady-state currents in the presence of CaM or CaM12 (Figure 1A–C, Figure 1—figure supplement 1). In contrast, the response was attenuated or precluded in the presence of CaM1234 or CaM34 (Figure 1A–C). Because structural and functional studies suggested a critical role of EF3 (Chang et al., 2018; Kang et al., 2020; Sun and MacKinnon, 2017; Tobelaim et al., 2017; Zhuang and Yan, 2020), we tested the effect of CaM3 and CaM124. Whereas the H_2_O_2_ response in the presence of CaM124 (Figure 1A–C) was maintained, it was diminished with CaM3 (Figure 1D). Importantly, while the response to H_2_O_2_ was abolished in the presence of CaM3, another K_V_7 activator, retigabine, still produced strong activation of K_V_7.4 current under these conditions (Figure 1D). Retigabine activates K_V_7 channels by binding to a hydrophobic pocket between S4 and S5 domains, a site that does not overlap with CaM binding site (Wuttke et al., 2005). These results suggest that EF3 of CaM is necessary for augmentation of K_V_7 channels by H_2_O_2_ specifically.

**Figure 1—figure supplement 1.**
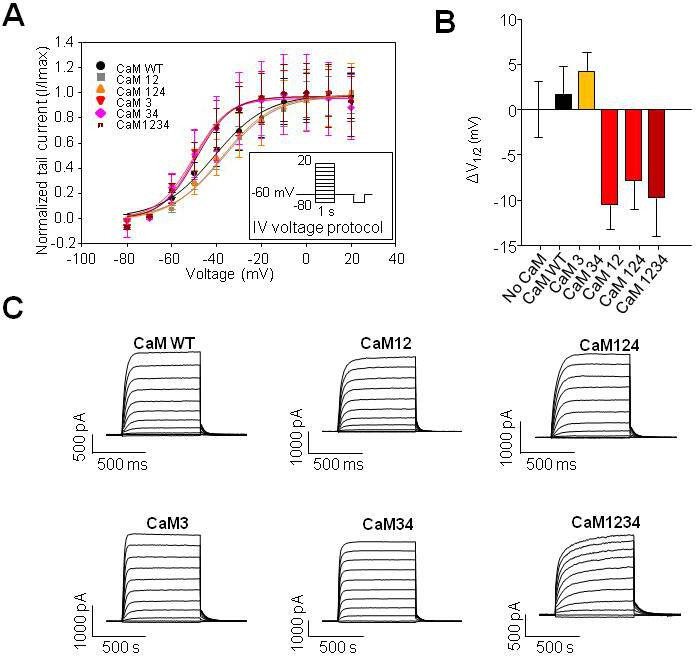
The current voltage relationship of cells transfected with mutant CaM does not differ significantly from CaMWT. (**A**) Current voltage relationship of K_V_4 and CaM transfected cells prior to treatment with H_2_O_2_. IV calculated through measuring tail current normalized to the maximal current in each condition (I/Imax). (**B**) Change in voltage at which half maximal current is produced (ΔV_1/2_) compared to CaMWT. (**C**) Representative IV traces for each CaM condition. Data presented is mean ± SEM, statistical evaluation by independent measures ANOVA with Dunnett’s post hoc *p<0.05. CaMWT n=6, CaM12 n=9, CaM124 n=8, CaM3 n=9, CaM34 n=6, and CaM1234 n=6.

**Figure 1—figure supplement 2.**
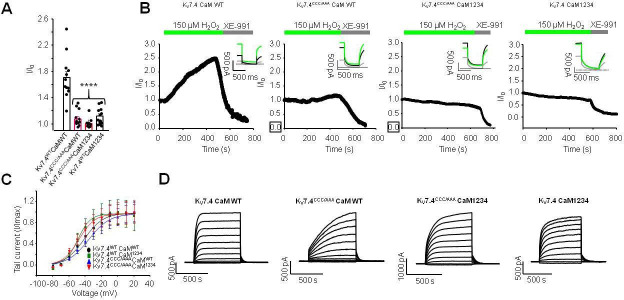
Further support for the link between H_2_O_2_, CaM, and the S2S3 linker in K_V_7 channels. (**A**) Response of K_V_7.4 or K_V_7.4 triple cysteine to alanine mutant (K_V_7.4CCC/AAA) to 150 µM H_2_O_2_ when co-transfected with CaMWT or CaM1234. (**B**) Representative current trains at –60 mV in response to 150 µM H_2_O_2_ followed by 10 µM XE-991. Inset: representative current traces from each condition. (**C**) Current voltage relationship of K_V_7.4 or K_V_7.4CCC/AAA and CaMWT or CaM1234 transfected cells prior to treatment with H_2_O_2_. IV calculated through measuring tail current normalized to the maximal current in each condition (I/Imax). (**D**) Representative IV traces for each condition. Data presented are mean ± SEM, statistical evaluation by independent measures ANOVA with Dunnett’s post hoc ****p<0.0001. Figure 1—figure supplement 2—source data 1.Link between H_2_O_2_, CaM and the S2S3 linker in K_V_7 channels.

EF-hand mutations used above mimic Ca^2+^-free (apo) state of the CaM, with CaM1234 being completely Ca^2+^-free, while other mutants are partially Ca^2+^-free. Since CaM1234 prevented the K_V_7.4 current augmentation by H_2_O_2_ (as did the other mutants containing EF3 mutation), we therefore tested if ‘sponging’ intracellular Ca^2+^ by pre-incubating the cells with BAPTA-AM also prevent the H_2_O_2_ effect on K_V_7.4. BAPTA-AM crosses the membrane and release the strong Ca^2+^ chelator BAPTA intracellularly, thereby lowering resting Ca^2+^ levels. The response to oxidation was indeed virtually abolished under these conditions.

As expected, the effect of H_2_O_2_ was absent after substituting the redox-sensitive triplet of cysteine residues at the positions 156, 157, and 158 in the S2S3 linker of K_V_7.4 by alanine residues (Figure 1—figure supplement 2). There was no difference whether WT CaM or CaM1234 was present; in either case, the current produced by CCCAAA K_V_7.4 was only marginally affected by 150 µM H_2_O_2_ (Figure 1—figure supplement 2). Interestingly, all CaM mutants containing EF3 mutations (CaM3, CaM34, and CaM1234) produced small negative shift in K_V_7.4 voltage dependence (Figure 1—figure supplement 1), a finding consistent with a presumed removal of a tonic inhibitory effect of calcified EF3.

Overall, these experiments suggested that EF3 of CaM and cysteine residues in the S2S3 of K_V_7.4 are necessary for current activation by H_2_O_2_. We hypothesize that binding of Ca^2+^ to EF3 partially inhibits K_V_7.4; preventing binding or removing Ca^2+^ from this location disinhibits the channel. We further hypothesize that oxidative modification of S2S3 cysteine residues antagonizes the EF3/Ca^2+^ inhibition of K_V_7.4. Yet, the interpretation of this effect requires caution since CaM over-expression also affects the number of the channels at the plasma membrane (Etxeberria et al., 2008; Gomis-Perez et al., 2017). To get further insights, we analyzed the behavior of the isolated CRD, without constrains imposed by other channel domains, the membrane, or the complexity of potential intracellular signaling cascades evoked in vivo.

### CaM remains attached to the AB fork

To test the stability of CaM engagement in the CRD, a fluorescent tag was placed in CaM (CaM-YFP) and another in the K_V_7.2 AB fork (mTFP1-AB). Two complementary assays were performed. In the first one, the complex was established with both components carrying a fluorescent tag, yielding an initial significant FRET value. Then, the complex was incubated up to 3 hr with excess of CaM (10-fold), devoid of any tag. Here, exchange between anchored and free CaM should be accompanied by a reduction in FRET. In the second assay, the complex was established between a tagged AB fork and label-free CaM. Subsequently, the complex was incubated with fluorescently tagged CaM. Exchange of anchored and free CaM should be accompanied by an increase in FRET. No changes in FRET were observed after 3 hr, neither in the presence nor in the absence of free Ca^2+^ (n=4; Figure 2—figure supplement 1). Thus, under these in vitro conditions, CaM remained firmly attached to the AB fork. Incidentally, the magnitude of FRET changes between mTFP1-AB, tagged just before helix A, in complex with CaM-YFP, tagged at the C-lobe, are best described as the C-lobe remaining bound to helix A in the presence of Ca^2+^, as previously revealed by NMR (Nuclear Magnetic Resonance) analysis of the complex (Bernardo-Seisdedos et al., 2018).

### Ca^2+^ binding to EF3 is critical for signaling

**Figure 2.**
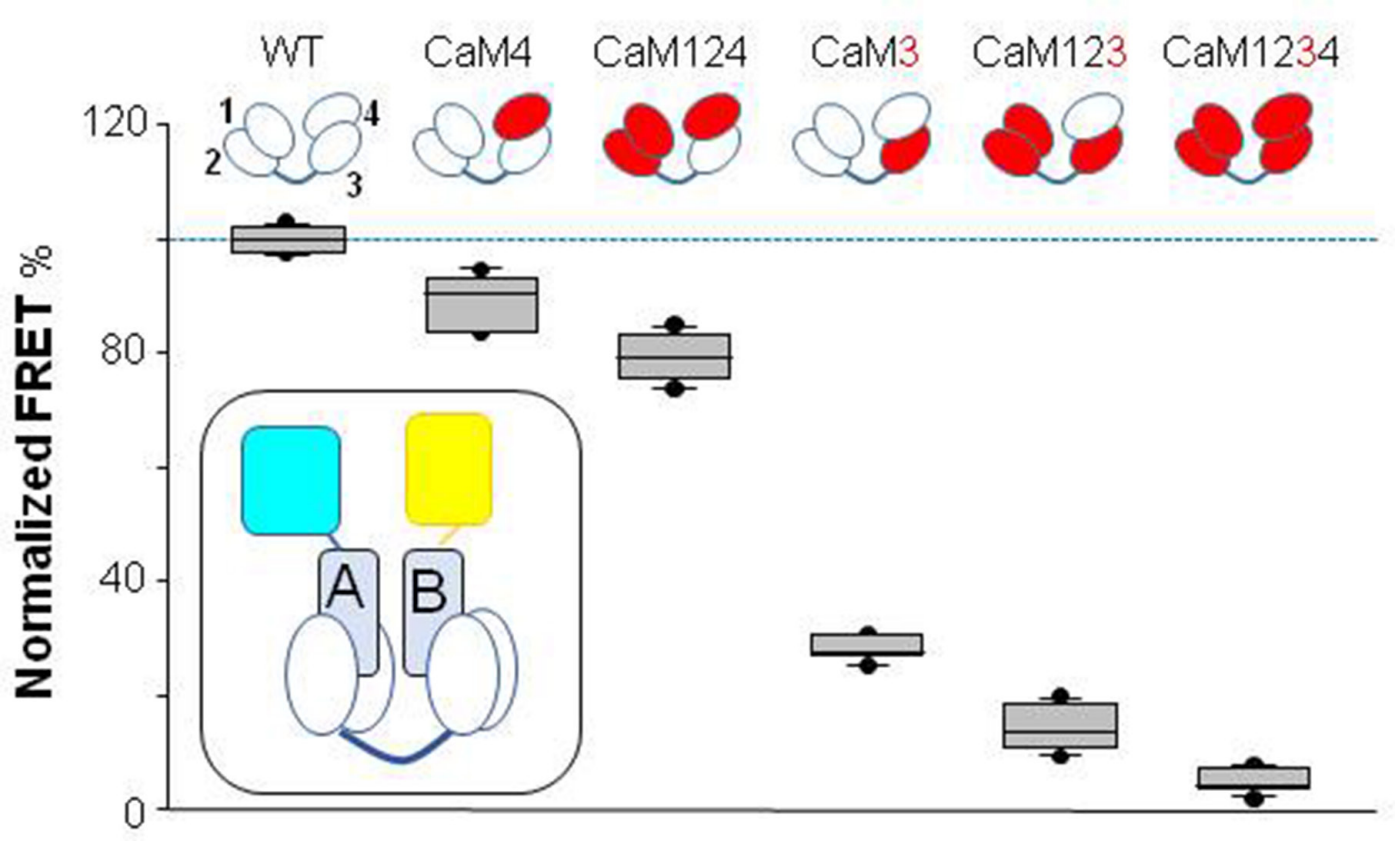
Influence of Ca^2+^-binding abolishing mutations in EF hands on Ca^2+^-dependent FRET changes. Top: cartoon representation of CaM mutants. The EF-hands carrying a mutation that preclude Ca^2+^ binding are colored in red. Bottom: box-plot of the relative FRET index change produced by Ca^2+^ for the AB fork in complex with the indicated mutated CaM. Note that in the complex with CaM3 and CaM123, the changes prompted by 16 µM Ca^2+^ were almost obliterated, whereas in the complex with CaM124 the response was preserved. Each plot represents the average of six independent experiments. FRET index was defined as the ratio of the fluorescence peak between mcpVenus (yellow acceptor) and mTFP1 (blue donor). The index was normalized to the value obtained with WT CaM. Experiments were performed at 500 nM of *h*AB:CaM purified complex, in a 1:1 ratio. Inset: cartoon representing the FRET sensor in complex with CaM (mTFP1-hA-hB-Venus/CaM). Figure 2—source data 1.Tabulated FRET values for each condition.

Wild-type or mutant CaMs were co-expressed with the K_V_7.2 CRD in bacteria, the 1:1 complex was purified, and Ca^2+^ signaling was examined by monitoring the transfer of energy between the two fluorophores attached to the N- and C-termini of the AB fork with a flexible linker (see inset in Figure 2). This flexibility favors that FRET efficiency would be mainly proportional to packing of hA and hB. FRET efficiency was reduced in a Ca^2+^ concentration-dependent manner as previously described (Bernardo-Seisdedos et al., 2018). Mutations into EF1 and EF2 (CaM12) did not significantly alter Ca^2+^-dependent signaling (n=6), whereas mutations at either EF-hands 3 or 4 (CaM3 or CaM4) reduced the magnitude of FRET changes (n=6). The extent of the effect was significantly decreased in the complex with CaM3, with a minor effect in the complex with CaM4 (Figure 2).

**Figure 2—figure supplement 1.**
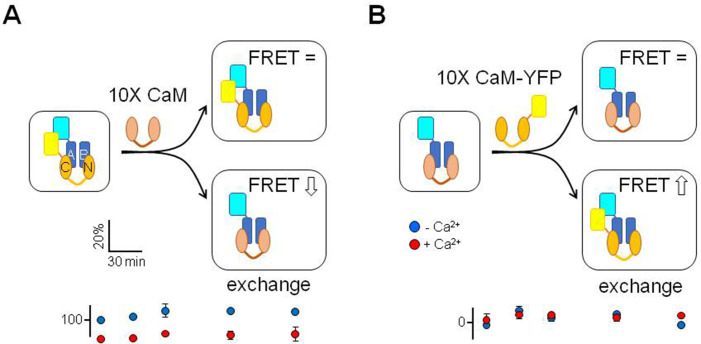
Residence of CaM in the calcium responsive domain (CRD) complex. Top: cartoon representation of the assays. A blue fluorescent protein was attached to the AB fork. In one experiment (**A**), the tagged AB fork was complexed with CaM tagged with a yellow fluorescent protein. Subsequently, the complex was incubated with excess CaM, devoid of any tag (n=4). A reduction in FRET over time is expected if there was CaM exchange. In another experiment (**B**), the fork was complexed with CaM and mixed with excess CaM tagged with a yellow fluorescence protein (n=4). Increase in FRET is expected if CaM exchange takes place. Bottom: FRET values over time in the presence and absence of Ca^2+^. Figure 2—figure supplement 1—source data 1.Residence of CaM in the CRD complex.

**Figure 2—figure supplement 2.**
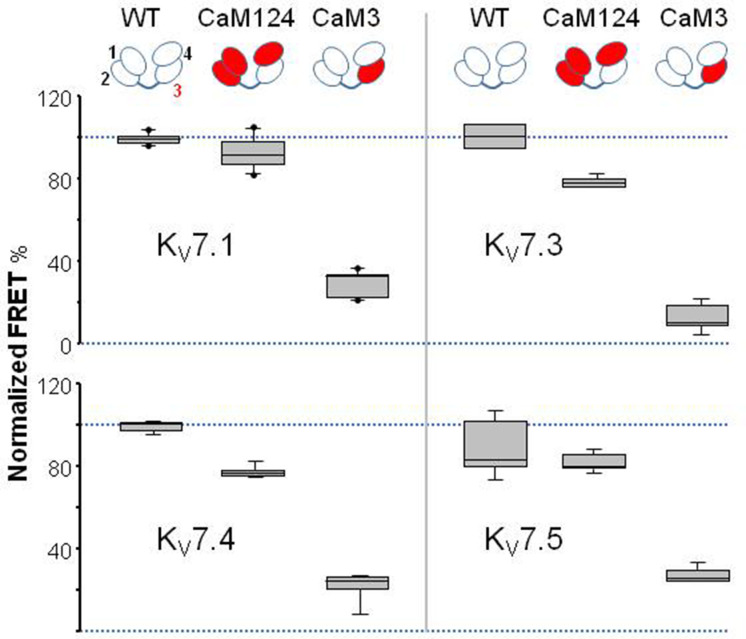
Box plot of the influence of Ca^2+^-binding abolishing mutations in EF hands on Ca^2+^-dependent FRET change on the indicated K_V_7 isoforms. Each plot represents the average of at least six independent experiments. Figure 2—figure supplement 2—source data 1.Tabulated FRET values for each condition.

The role of EF3 was further examined combining Ca^2+^-binding canceling mutations in EF1, EF2, and EF4-hands. The AB/CaM124 complex, that is, with only EF3 able to bind Ca^2+^, presented a response to Ca^2+^ that was ~80% that of the AB in complex with WT CaM (n=6). In contrast, the response of the complex with CaM3 was reduced to ~30% (n=6). A similar strategy was followed to evaluate the role of the EF4 hand, testing complexes with CaM123 and CaM4. In the complex with CaM123, the response was almost abolished, whereas in the complex with CaM4, the response was about 90% of that of WT (n=6; Figure 2). Similar results were obtained with complexes between the CDRs of the remaining K_V_7 family members and mutant CaM (Figure 2—figure supplement 2). Thus, EF3 plays a significant role in transmitting Ca^2+^ signals to the AB fork, and EF4 plays a secondary function.

### Peptides derived from the K_V_7 S2S3 loop interact with CaM

A subset of cryo-EM K_V_7.1 channel particles has revealed a likely interaction between the S2S3 loop of the channel voltage sensor and the EF3 of CaM (Sun and MacKinnon, 2017; Sun and MacKinnon, 2020; Figure 3—figure supplement 1), which, in turn, is engaged to the AB fork. Similar arrangements were reported for the K_V_7.2 CRD (Bernardo-Seisdedos et al., 2018). These structural studies suggest that the privileged role of EF3 may derive from constrains imposed by the channel architecture. To address the significance of this interaction in the absence of other channel domains, changes in the fluorescent emission of dansylated CaM (D-CaM) produced by peptides derived from the K_V_7 S2S3 sequence were monitored (Alaimo et al., 2013; Supplementary file 1). Interaction of alpha helices within the groove of the CaM lobes results in an increase in fluorescent emission of D-CaM, whereas the binding of Ca^2+^ to the EF-hands causes, in addition to an increase in fluorescence, a leftward shift in the position of the peak in the emission spectrum (Alaimo et al., 2013).

**Figure 3.**
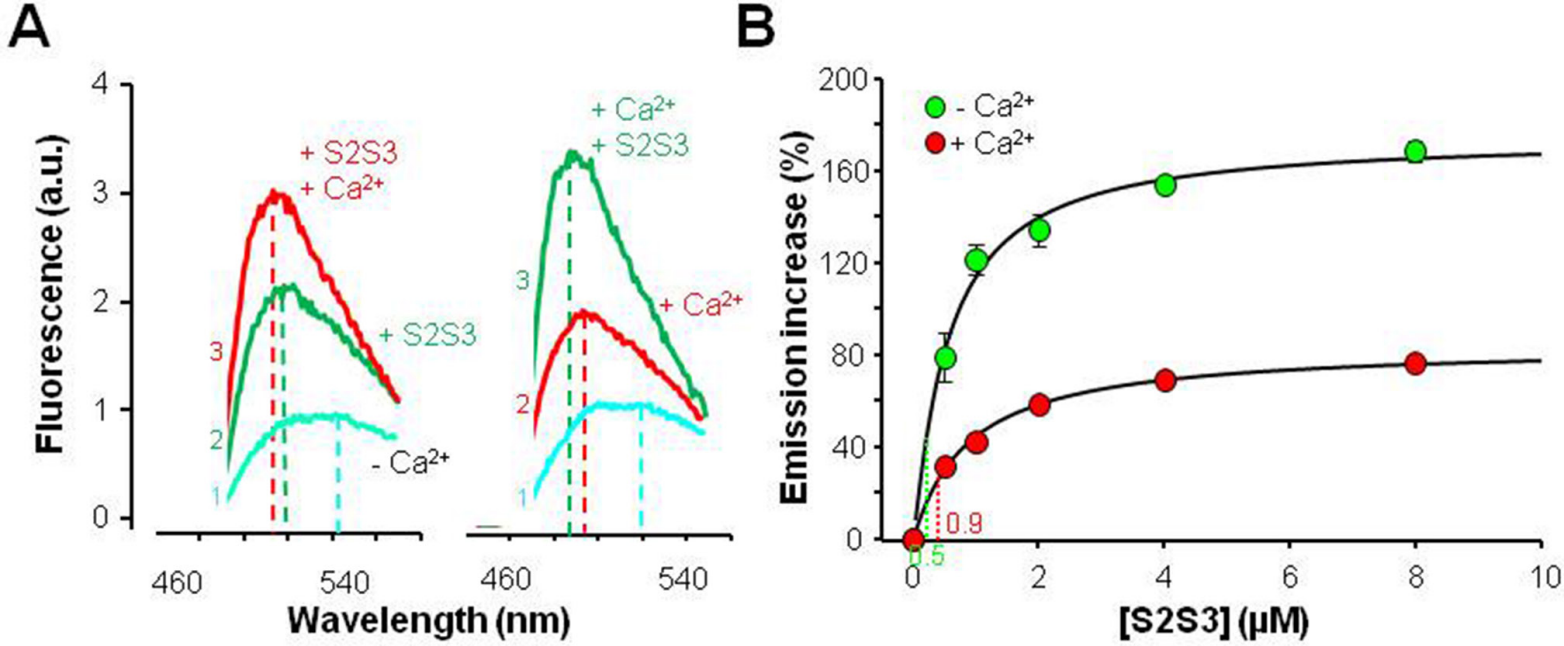
Effects of a 24 residues K_V_7 S2S3 peptide on fluorescence emission of dansylated calmodulin (D-CAM). (**A**) Emission spectra of D-CaM (50 nM) in Ca^2+^-free conditions (cyan), and after subsequent sequential addition of the S2S3 peptide (16 µM, green), and Ca^2+^ (10 µM free concentration, red). The order of additions is indicated at the left of each trace. (**B**) Dose-dependent relative fluorescent emission increase as a function of S2S3 peptide concentration, in the absence (green) and the presence of Ca^2+^ (10 µM, red). For this purpose, the maximum fluorescence D-CaM emission was measured between 490 and 500 nm and normalized with respect to the reference value (D-CaM with no added Ca^2+^ [green] and D-CaM with 10 µM free Ca^2+^ [red]). A Hill equation was fitted to the data (continuous line) with EC_50_=0.88 ± 0.12 and 1.63±0.07 µM, in the absence and the presence of Ca^2+^, respectively. The K_V_7.1 S2S3 peptide sequence was Ac-RLWSAGCRSKYVGVWGRLRFARK-NH_2_. Figure 3—source data 1.Spectra data for the indicated conditions, and tabulated peak values.

The response to S2S3 peptides rendered an analogous profile to that of Ca^2+^: a leftward shift on the emission peak and an increase in fluorescent emission (Figure 3). A similar response was observed when S2S3 peptides derived from the sequence of human K_V_7.1 thought K_V_7.5 were tested (n=3; Figure 3—figure supplement 2). The relative increase in emission intensity was twice as large in the absence of Ca^2+^ (Figure 3). This is in contrast to what has been observed for peptides or targets that are embraced within the CaM lobes, in which the relative increase is similar with and without Ca^2+^ (Alaimo et al., 2014; Bonache et al., 2014). Interestingly, the leftward shifts caused by Ca^2+^ and the peptide were additive (Figure 3). These results suggest that Ca^2+^ and the peptide can interact with CaM simultaneously and that Ca^2+^ mitigates the effect of the peptide on D-CaM.

**Figure 3—figure supplement 1.**
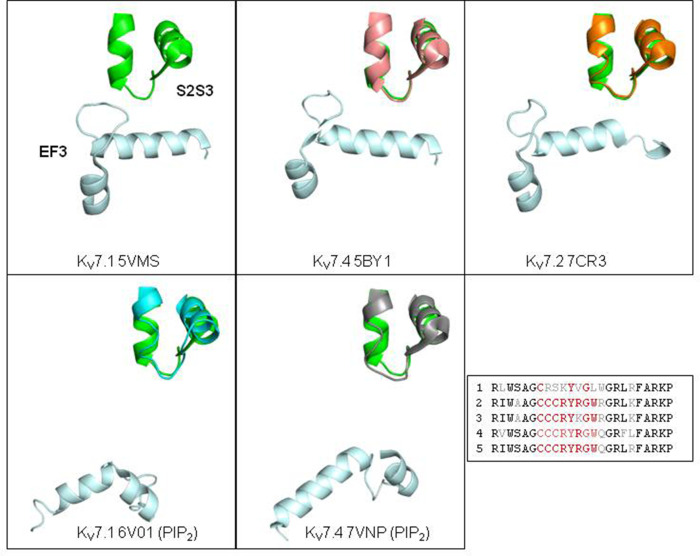
Relative disposition of calmodulin EF3 and S2S3 loop from different K_V_7 channel/CaM complexes solved by cryo-EM. The PDB of each structure is indicated at the bottom of each panel. The 5VMS structure of K_V_7.1 suggests a potential interaction between the S2S3 loop and the EF3 hand, whereas this interaction is not apparent in cryo-EM images from other K_V_7 channels, or K_V_7.1 channels solved in the presence of PIP_2_ (PDB 6V01). The structure S2S3 of K_V_7.1 PDB 5VMS in green is superimposed in each figure. The main differences appear located at the distal region of the proximal helix of the S2S3 hairpin and the loop in the K_V_7.4 structure (PDB 7VNP), which was trapped in an open channel configuration thanks to the presence of PIP_2_, whereas it is remarkably similar in the structure of K_V_7.4 (PDB 5BY1) trapped in a close conformation. Inset: alignment of the S2S3 sequences.

**Figure 3—figure supplement 2.**
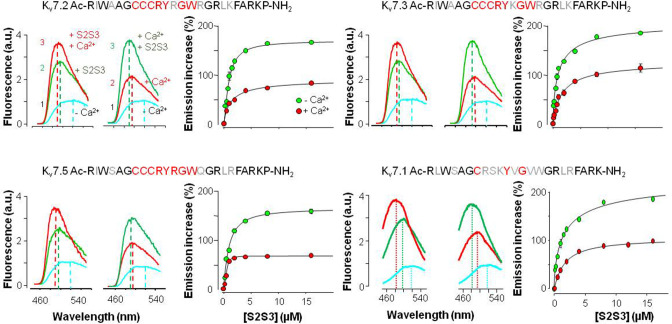
Effect of K_V_7 S2S3 peptides on fluorescence emission of dansylated calmodulin (D-CaM). The residues forming the loop are highlighted in red in each peptide sequence. Emission spectra of D-CaM (50 nM) in Ca^2+^-free conditions (cyan), and after subsequent sequential addition of the S2S3 peptide (16 µM, green), and Ca^2+^ (10 µM free concentration, red). The order of additions is indicated at the left of each trace in the panel at the top left. A Hill equation was fitted to the emission increase data (continuous line) with EC_50_=0.89 ± 0.04 and 1.37±0.23 µM, (n=3) in the absence and presence of Ca^2+^, respectively for K_V_7.2 S2S3, 1.04±0.12 (n=3) and 1.78±0.34 µM for K_V_7.3 (n=3), 0.56±0.06 and 0.89±0.07 µM for K_V_7.4 (n=3), and 0.88±0.12 and 1.63±0.07 µM for K_V_7.1 (n=3).

### NMR reveals interaction of the S2S3 peptide with the C-lobe of the AB/CaM complex

**Figure 4.**
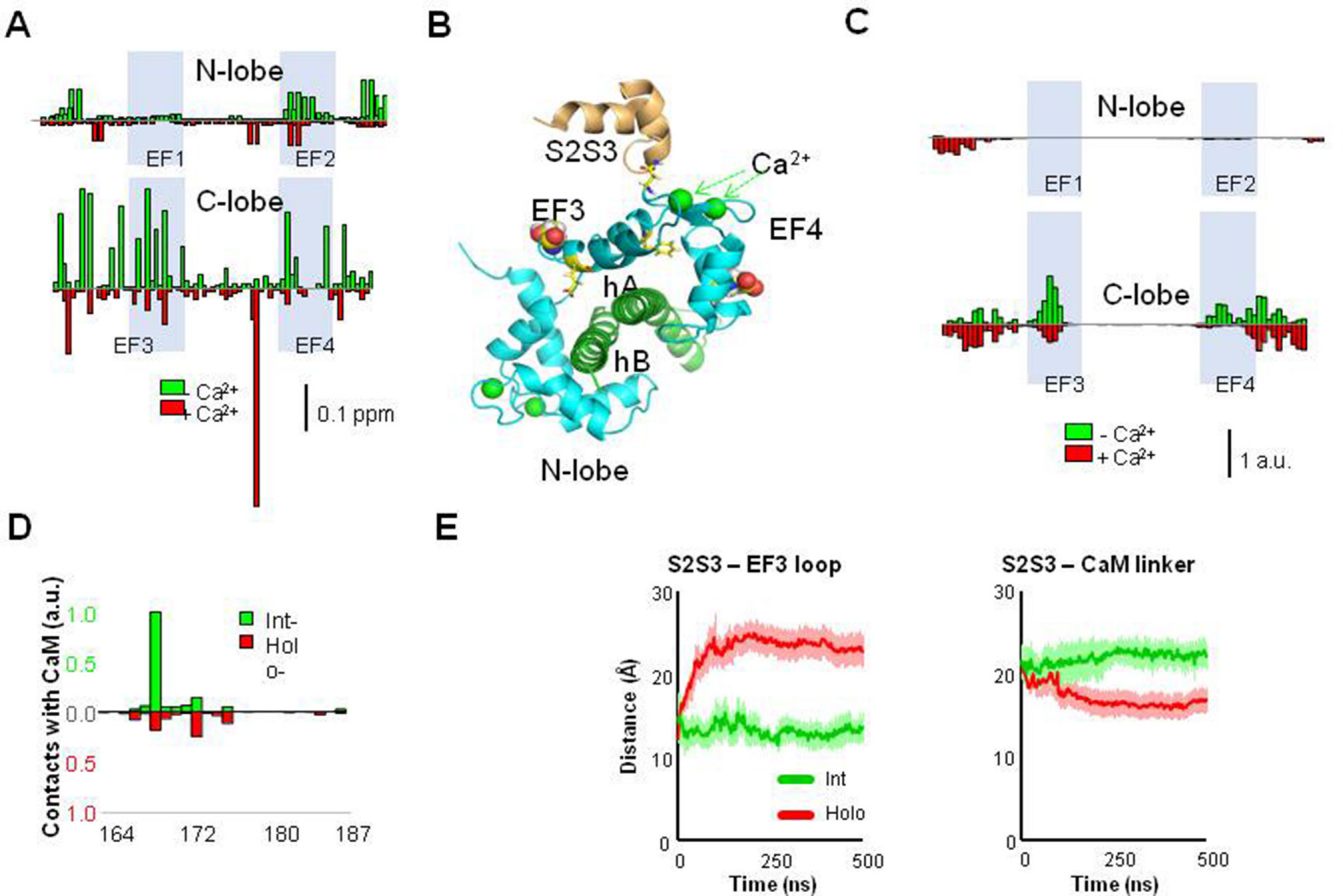
Interaction between the K_V_7 S2S3 peptide and CaM in complex with K_V_7.2 CDR. (**A**) The chemical shift perturbation (CSP) analysis shows that the magnitude of local residue environmental alterations detected by NMR is larger in the C-lobe, both in the presence and in the absence of Ca^2+^. (**B**) Structural mapping of the main CSPs in the presence of Ca^2+^ over Ca^2+^-loaded K_V_7.2 CaM/CDR complex. The two resides with the larger displacements are represented as balls, whereas the remaining above three times the mean are represented as sticks. The structure of the S2S3 loop was derived from the Cryo-EM PDB 5VMS (Sun and MacKinnon, 2017) and placed according to structural alignment of the C-lobe of PDB 6FEH (Bernardo-Seisdedos et al., 2018). (**C**) Contact map derived from molecular dynamic (MD) simulations of the S2S3/CaM complex. Normalized CaM contacts with the S2S3 peptide residues (10 Å cut-off) for int- (green) and holo-systems (red; see Figure 5—figure supplement 1). Vertical calibration bar is in arbitrary units (a.u.). (**D**) S2S3 contact map with CaM residues (4 Å cut-off; see Figure 5—figure supplement 1). (**E**) Distance as a function of time between the mass centers of the EF3 loop (residues D93-G98) and (i) the S2S3 loop (residues R164-L173; left) or (ii) the linker connecting CaM lobes (residues R74-E84; right). Bars indicate SEM (n=6). Figure 4—source data 1.Tabulated data values for NMR chemical shift perturbations.Tabulated contact maps between calmodulin and Kv7.1 S2S3, and tabulated distance between S2S3 and EF3 mass centers. Figure 4—source data 2.NMR raw spectra of KV7.2/Calmodulin complex with and without calcium in presence of S2S3 peptide. Figure 4—source data 3.Replica 1: Molecular dynamic trajectory of holo system, calcified calmodulin in presence of S2S3. Figure 4—source data 4.Replica 2: Molecular dynamic trajectory of holo system, calcified calmodulin in presence of S2S3. Figure 4—source data 5.Replica 3: Molecular dynamic trajectory of holo system, calcified calmodulin in presence of S2S3. Figure 4—source data 6.Replica 4: Molecular dynamic trajectory of holo system, calcified calmodulin in presence of S2S3. Figure 4—source data 7.Replica 5: Molecular dynamic trajectory of holo system, calcified calmodulin in presence of S2S3. Figure 4—source data 8.Replica 6: Molecular dynamic trajectory of holo system, calcified calmodulin in presence of S2S3. Figure 4—source data 9.Replica 6: Molecular dynamic trajectory of holo system, calcified calmodulin in presence of S2S3. Figure 4—source data 10.Replica 1: Molecular dynamic trajectory of int system, calcified N-lobe (no calcium C-lobe) of calmodulin in presence of S2S3. Figure 4—source data 11.Replica 2: Molecular dynamic trajectory of int system, calcified N-lobe (no calcium C-lobe) of calmodulin in presence of S2S3. Figure 4—source data 12.Replica 3: Molecular dynamic trajectory of int system, calcified N-lobe (no calcium C-lobe) of calmodulin in presence of S2S3. Figure 4—source data 13.Replica 4: Molecular dynamic trajectory of int system, calcified N-lobe (no calcium C-lobe) of calmodulin in presence of S2S3. Figure 4—source data 14.Replica 5: Molecular dynamic trajectory of int system, calcified N-lobe (no calcium C-lobe) of calmodulin in presence of S2S3. Figure 4—source data 15.Molecular dynamic trajectory of S2S3 peptide y solution.

The NMR signals from labeled WT CaM complexed with non-labeled K_V_7.2 AB fork were compared in the presence and absence of the S2S3 peptide and with Ca^2+^ added (holo-CaM, four EF-hands Ca^2+^-loaded) or not added (int-CaM, N-lobe Ca^2+^-loaded). Chemical shift perturbations (CSPs) produced by the S2S3 peptide (13 equivalents) in the ^1^H-^15^N-HSQC map of int-CaM (holo-N-lobe and apo-C-lobe) and holo-CaM in complex with the K_V_7.2 CRD are shown in Figure 4A (see also Figure 4—figure supplement 1). In the presence of the S2S3 peptide, several resonances of CaM residues in the spectrum were shifted, most of them located in the C-lobe. The CSPs perturbations, color-coded in the structure of the human K_V_7.2 CRD in Figure 4—figure supplement 1, are consistent with the S2S3 loop interacting predominantly with the EF3 loop, both in absence and in the presence of Ca^2+^. EF3 displacements were observed for D94, N98, Y100, I102, and A104, whereas for EF4, changes in the environment of I131 and E139 are beyond the threshold level (Figure 4B). Thus, Ca^2+^ addition produces a significant perturbation map, which is in line with the differential relative increase in fluorescence caused by the peptide in the D-CaM assay (Figure 3B). Next, we performed atomistic molecular dynamics (MDs) simulations to investigate the interactions between the K_V_7.1 S2S3 peptide and int- or holo-CaM in complex with the K_V_7.2 CRD (Supplementary file 2). Consistent with the NMR interaction experiment, the contact map obtained from the simulations shows that the peptide interacts mainly with the EF3 loop and the linker connecting CaM lobes (Figure 4C–D and Figure 4—figure supplement 2). In contrast, there were no contacts in the region connecting EF3 and EF4, suggesting that the CSPs observed are better interpreted as an allosteric effect, rather than a direct contact with the peptide.

**Figure 4—figure supplement 1.**
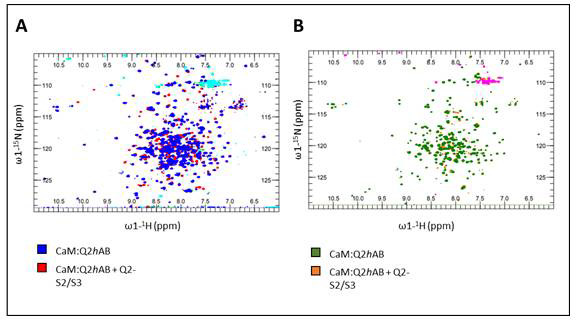
^15^N-HSQC of K_V_7.2 CDR:CaM titrated with unlabeled K_V_7.1-S2S3 in absence (left) and in presence of 1 mM Ca^2+^. (**A**) ^15^N-HSQC of K_V_7.2 CDR:CaM complex at 75 µM (blue) and with K_V_7.1-S2S3 peptide at 1 mM (red). Signal overlap demonstrates that the structure of the complex was not altered in the presence of the peptide. (**B**) Green spectra corresponds to the complex K_V_7.2 CDR:CaM with 1 mM Ca^2+^, and orange to K_V_7.2 CDR:CaM 1 mM Ca^2+^ and 1 mM K_V_7.1-S2S3 peptide. Figure 4—figure supplement 1—source data 1.HSQC of KV7.2 CDR:CaM titrated with unlabeled KV7.1-S2S3 in absence of 1 mM Ca2+.

**Figure 4—figure supplement 2.**
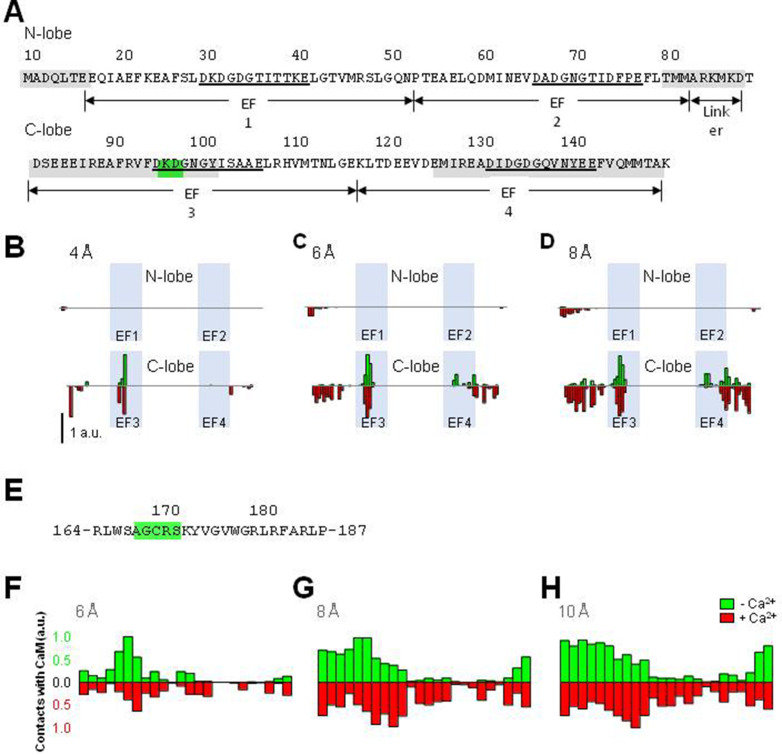
Contact maps derived from molecular dynamic (MD) simulations of the S2S3/CaM complex. (**A**) CaM sequence highlighting the most relevant residues for the contacts with S2S3 peptide with a cut-off at 10 Å (in gray) and at 4 Å (in green). The EF loops are underlined. (**B**) Normalized CaM contacts between CaM and S2S3 peptide residues with cut-off at 4 Å, (**C**) 6 Å, and (**D**) 8 Å, for int- (green) and holo-systems (red). (**E**) S2S3 peptide sequence highlighting in green the most relevant residues for the contact with CaM with a cut-off at 4 Å. (**F**) Normalized S2S3 peptide contacts between CaM and S2S3 peptide residues with cut-off at 6 Å, (**G**) 8 Å, and (**H**) 10 Å. Figure 4—figure supplement 2—source data 1.HSQC of KV7.2 CDR:CaM titrated with unlabeled KV7.1-S2S3 in presence of 1 mM Ca2+.

**Figure 4—figure supplement 3.**
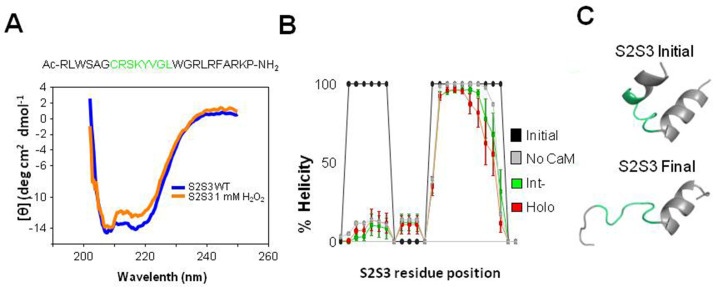
CD spectra of S2S3 peptide before and after treatment with H2O2. (**A**) Circular Dichroism of the K_V_7.1 S2S3 peptide. The residues forming the loop are highlighted in green in the peptide sequence. CD (Circular Dichroism) spectra recorded at a fixed incubation time of the peptide (50 nM) in control (blue) and in presence of 1 m H_2_O_2_ (orange). Helicity (**H**) was 63.4%, S+T antiparallel β-sheet conformations was 16.2%, and U for disordered structures were estimated to be 21.1%. In the presence of 1 mM H_2_O_2_, H=63.6%, S+T = 15.3%, and U=21.8%. Spectrum is the average of 40 runs. (**B**). Percentage of the S2S3 helical character relative to the whole trajectory. Bars indicate SEM (n=6). Helical character of the initial S2S3 structure is shown in black. The peptide in solution (gray line) adopts a similar structure regardless of the presence or the absence of the CaM/K_V_7.2 complex. Both 3_10_ and α-helices were combined to compute the degree of helicity. (**C**) Representative structures of the S2S3 peptide at initial (left) and final (right) times. Loop residues are shown in green. Figure 4—figure supplement 3—source data 1.Raw data for CD spectra of S2S3 peptide before and after treatment with H2O2, and tabulated values of percentage helicity vs position.

Regarding the S2S3 peptide, residues that form an intracellular loop located between W166 and G176 are the ones that interact predominantly with CaM (Figure 4D). During the course of the simulation, the C-terminal region adopted an α-helix conformation for ≥97.8% of the time (see Figure 4—figure supplement 3). The N-terminal that started as a 3_10_ helix became unstructured after the initial equilibration. It is reasonable to expect such differential stability since the N- and C-helices were initially formed by 6 and 10 residues, respectively, and 3_10_ helices are less stable than α-helices (Bolin et al., 1999).

The interaction between S2S3 and EF3 was more stable when it was not loaded with Ca^2+^ (Figure 4F, left). In contrast, the main contacts of the holo-system were established primarily with the linker connecting CaM lobes (Figure 4F, right). To analyze the interaction between S2S3 and EF3, we measured the distance between the center of mass of EF3 and S2S3 loops or the linker connecting the CaM lobes. The results suggest that the interaction between the S2S3 and the empty EF3 loops is rather stable, whereas Ca^2+^ occupancy prompts the movement of the peptide away from EF3 toward the linker on the lobes (Figure 4E). Thus, Ca^2+^ occupancy has an important influence on the S2S3/CaM interaction. These observations could fully explain the reduced CaM/S2S3 affinity in the presence of Ca^2+^ (see Figure 3).

### Reversal of Ca^2+^-EF3 signaling by S2S3 peptides

**Figure 5.**
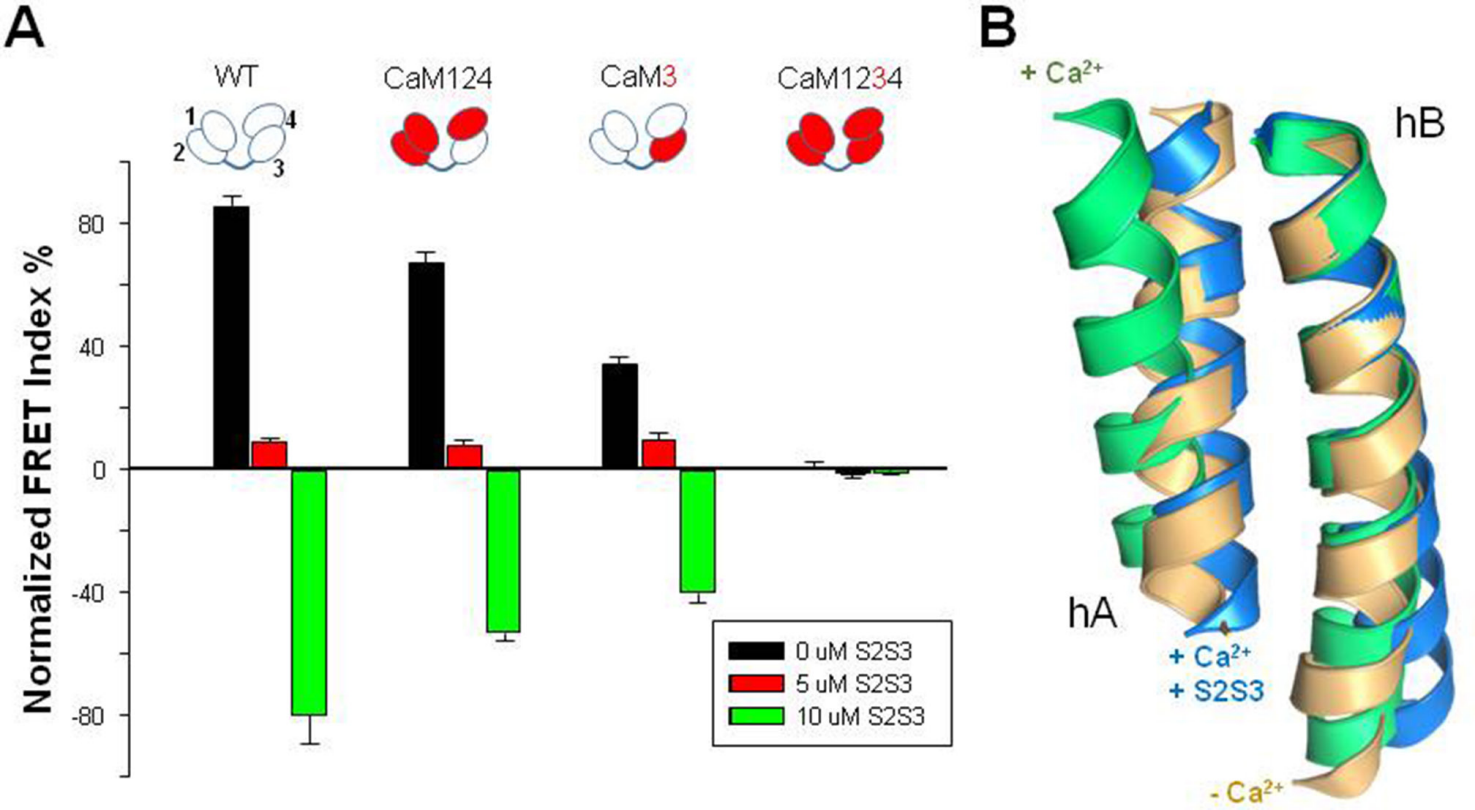
Relative FRET changes of the human K_V_7.2 in complex with mutant CaM. (**A**) FRET change after Ca^2+^ addition (10 µM) in the presence of the indicated concentrations of the S2S3 peptide: control (black), 0 µM S2S3, 5 µM S2S3 (red), and 10 µM S2S3 (green) (n=4). (**B**) Superposition of helices A and B solved in the absence of Ca^2+^ (gold, PDB 6FEH, K_V_7.2), in the presence of Ca^2+^ (green, PDB 6FEG, K_V_7.2), and interacting with the S2S3 loop in the presence of Ca^2+^ (blue, PDB 5VMS, K_V_7.1). Figure 5—source data 1.Tabulated FRET values for each condition.

Changes on FRET index in response to Ca^2+^ in the presence of S2S3 peptides were monitored as previously described (see Figure 2). Figure 5 shows that the Ca^2+^-dependent reduction in FRET index was mitigated as the concentration of peptide was increased. At high-peptide concentrations (≥10 µM), the FRET index increased, suggesting that the distance/orientation of AB helices was even more favorable than in the absence of Ca^2+^. A similar behavior was observed when the effect of the peptide for the other K_V_7 family members was examined (Figure 5—figure supplement 1).

**Figure 5—figure supplement 1.**
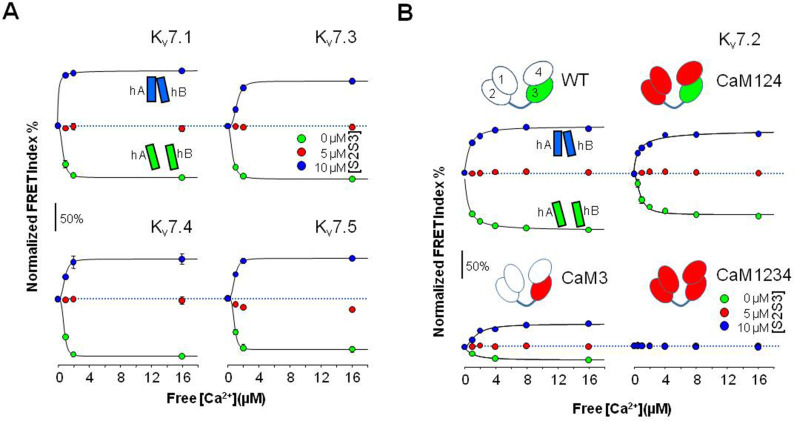
Relative FRET changes of K_V_7 CRD in complex with CaM. (**A**) Relative FRET changes of K_V_7 calcium responsive domain (CRD) in complex with CaM. The concentration of peptide employed is indicated by the color of the symbol. Ca^2+^ dose-response in the presence of the indicated concentrations of the S2S3 peptide, normalized to the maximal response. FRET changes expanded from 100% reduction to 70% increase. Dotted lines mark no changes in FRET. The continuous lines are the result of the fit of a Hill equation, with EC_50_ values of 0.70±0.04 µM, 0.93±0.03 µM, 0.86±0.1, and 0.85±0.33 µM for K_V_7.1, K_V_7.3, K_V_7.4, and K_V_7.5, respectively, in the absence of S2S3. In the presence of 10 µM S2S3, the EC_50_ values were 1.06±0.2 µM, 1.17±0.6 µM, 0.94±0.6 µM, and 0.89±0.33 µM for K_V_7.1, K_V_7.3, K_V_7.4, and K_V_7.5, respectively. Each plot represents the average of four independent experiments. (**B**) Ca^2+^ dose-response in the presence of the indicated concentrations of S2S3 peptide, normalized to the maximal response. FRET changes expanded from 100% reduction to 70% increase. Dotted lines mark no changes in FRET. The CaM mutant forming part of each complex is indicated on top of the corresponding dose-response curve. The continuous lines are the result of the fit of a Hill equation, with EC_50_ values of 0.84±0.04 and 0.83±0.07 µM in the absence of S2S3 for WT and CaM124 (n=4), respectively. In the presence of 10 µM S2S3, the EC_50_ values were 0.62±0.91, 0.62±0.16, and 1.48±0.22 µM for WT, CaM124, and CaM3, respectively (n=4). Figure 5—figure supplement 1—source data 1.Tabulated FRET values for each condition.

S2S3 is not canceling the effect of Ca^2+^ by competing or displacing this cation from its binding site. Instead, the response to Ca^2+^ in the presence of S2S3, in terms of FRET index, was in the opposite direction than when the peptide was absent. The magnitude of signaling reversal was similar in WT and CaM124 complexes, whereas it was reduced in complexes with CaM3 (Figure 5). Thus, the direction/orientation of the movements in the AB fork when EF3 is loaded with Ca^2+^ is reversed upon interaction with S2S3.

### Treatment with H_2_O_2_ reduces the effect of the S2S3 peptide

The S2S3 loop, which is highly conserved among K_V_7 channels, contains one (K_V_7.1) or three cysteine residues (K_V_7.2–K_V_7.5, Figure 3—figure supplement 1). The cysteine site mediates an increase in channel open probability in response to oxidizing conditions (Gamper et al., 2006). We tested the influence of oxidation by removing DTT (Dithiothreitol) from the buffer and including H_2_O_2_ to obtain a derivate that will be referred to as oxidized-S2S3. Contrary to the increase observed with S2S3, no changes in fluorescent emission of D-CaM were observed after addition of oxidized-S2S3 (Figure 6—figure supplement 1).

**Figure 6.**
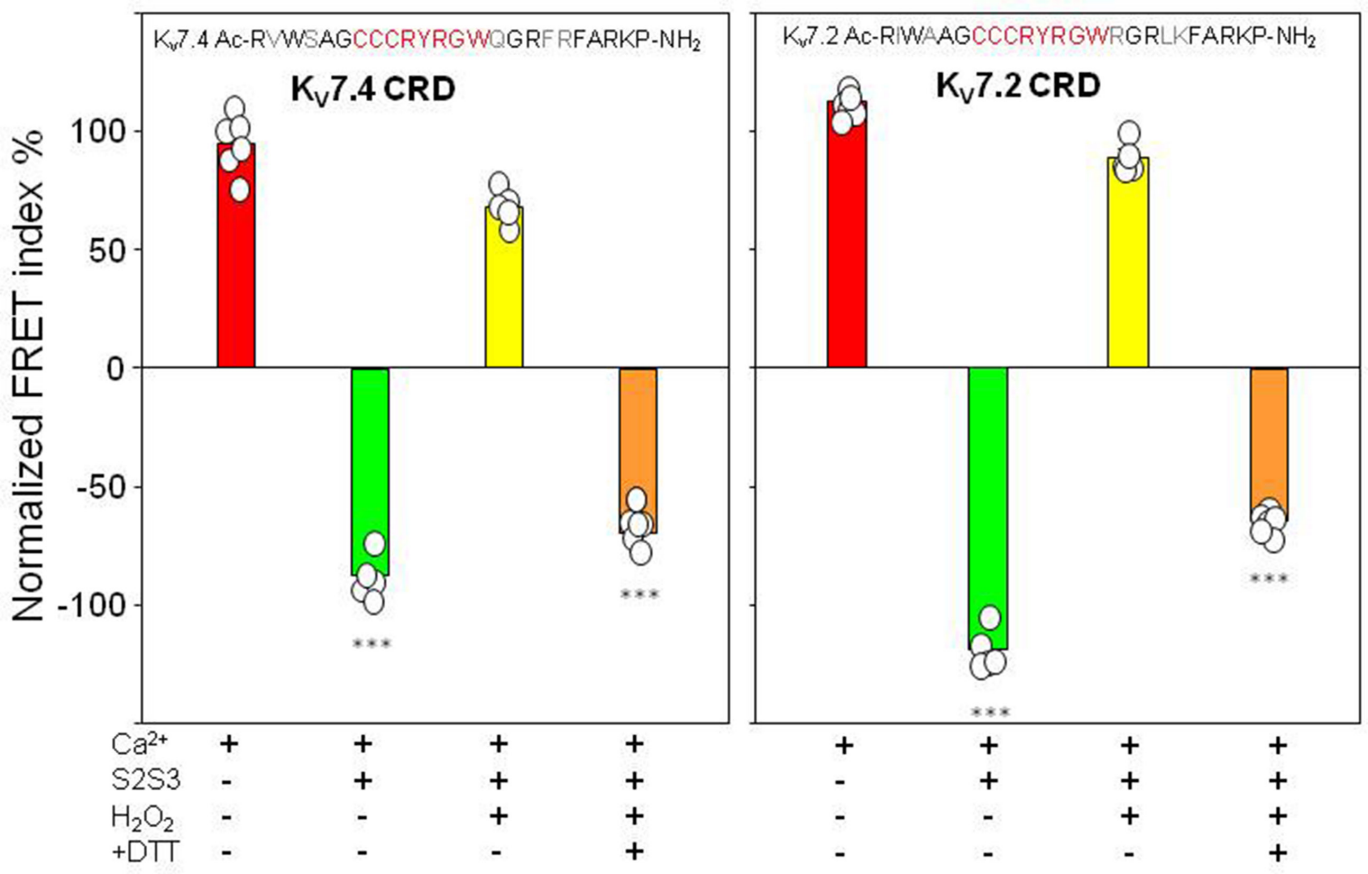
FRET efficiency changes prompted by oxidized and reduced S2S3 peptides. Difference in FRET efficiency in the absence and the presence of Ca^2+^. Left, K_V_7.4-S2S3 peptide and K_V_7.4 CRD. Right, K_V_7.2-S2S3 peptide and K_V_7.2 CRD. Similar results were obtained with K_V_7.1-S2S3 peptide (Figure 6—figure supplement 2). Red, sensor alone. Green, in the presence of 10 µM peptide. Yellow, with 10 µM oxidized peptide. Orange, oxidized peptide treated with 1 mM DTT. Bars represent mean ± SEM FRET-efficiency. *p<0.05; ***p<0.001. Each plot represents the average of at least six independent experiments. Figure 6—source data 1.Tabulated FRET values for each condition.

Treatment with H_2_O_2_ or DTT did not affect the response of the K_V_7.4AB/CaM or K_V_7.2AB/CaM complexes to Ca^2+^ (Figure 6—figure supplement 2). The Ca^2+^ titration profile using K_V_7.4AB/CaM or K_V_7.2AB/CaM complexes in the presence of oxidized K_V_7.4 or K_V_7.2 peptides (10 µM) was similar to that obtained in the absence of S2S3, suggesting that oxidized S2S3 can no longer affect the AB-CaM interaction (Figure 6). Using the K_V_7.4 sensor and oxidized K_V_7.4-S2S3 peptide, the relative FRET change in presence of Ca^2+^ was 68.22±6.90, i.e., a change qualitatively similar to that obtained in the presence of Ca^2+^ alone (94.84±5.93), and very different from that obtained with the untreated peptide (–87.50±8.26). The oxidized S2S3 peptide was incubated with the reducing agent DTT aiming to reverse the effect of the treatment with H_2_O_2_. We observed a partial recovery: after treating the oxidized peptide with DTT, the relative FRET change was similar to that observed with the untreated peptide (–69.45±12.80; Figure 6, left panel). Similar results were obtained using the K_V_7.2 sensor and the K_V_7.2-peptide (Figure 6, right panel) or when using the K_V_7.1-peptide (Figure 6—figure supplements 1 and 2).

**Figure 6—figure supplement 1.**
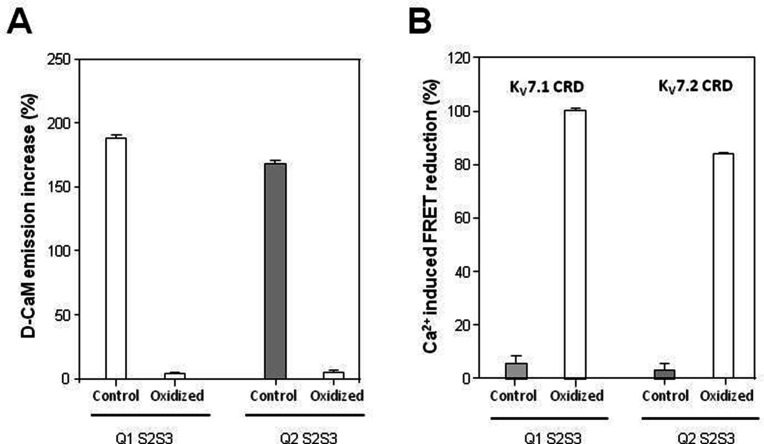
Fluorescent emission D-CaM enhancement caused by K_V_7.1 S2S3 and K_V_7.2 S2S3 peptides before and after oxidation. (**A**) Fluorescent emission dansylated calmodulin (D-CaM) enhancement caused by K_V_7.1 S2S3 and K_V_7.2 S2S3 peptides before and after oxidation. (**B**). Normalized FRET changes produced by 8 µM Ca^2+^, in the presence of 5 µM K_V_7.1 S2S3 and K_V_7.2 S2S3 peptides in mTFP-Q1hAB-Venus/CaM and mTFP-Q2hAB-Venus complexes, respectively, before and after oxidation. Bars represent the means ± SEM from at least six independent experiments. Figure 6—figure supplement 1—source data 1.Tabulated fluoresce emission values for each condition.

**Figure 6—figure supplement 2.**
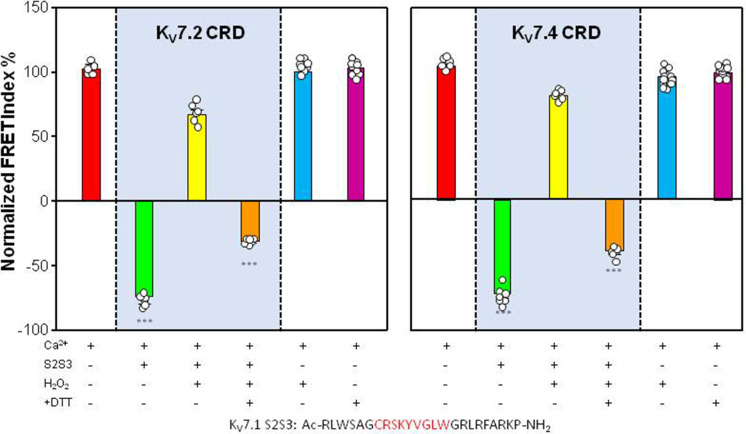
Percentage of FRET index changes prompted by oxidized and reduced S2S3 peptides. (**A**) Percentage of FRET index changes prompted by oxidized and reduced S2S3 peptides. Difference in % FRET index in the absence and the presence of Ca^2+^ with sensors derived from the K_V_7.4 and K_V_7.2 CRD sequences. Red, sensor alone. Green, in the presence of 10 µM K_V_7.1-S2S3 peptide. Yellow, with 10 µM oxidized peptide. Orange, oxidized peptide treated with 1 mM DTT. Blue, K_V_7.x/CaM complex treated with 1 mM H_2_O_2._ Purple, K_V_7.x/CaM incubated with 1 mM DTT. Bars represent mean ± SEM FRET-efficiency. *p<0.05; ***p<0.001. Each plot represents the average of at least six independent experiments. Bottom: sequence of the K_V_7.1-peptide. The residues forming the loop are highlighted in red in the peptide sequence. Figure 6—figure supplement 2—source data 1.Tabulated FRET values for each condition.

### All K_V_7 CRDs display a similar response to Ca^2+^

As mentioned above, the effect of S2S3 peptides having the sequence of each K_V_7 isoforms was tested in D-CaM assay, obtaining equivalent results for the set. The apparent affinity for every peptide was significantly lower in the presence of Ca^2+^ (Figure 3—figure supplement 2). A panel of K_V_7 biosensors in which the fork sequence was replaced by the equivalent segment from K_V_7.1, K_V_7.2, K_V_7.3, K_V_7.4, and K_V_7.5 human isoforms was created. FRET index was reduced for all biosensors in the presence of Ca^2+^, and the signal was significantly preserved in complexes formed with CaM124, whereas it was decreased in complexes with CaM3. The apparent affinity for Ca^2+^ was lower in the presence of the peptides, but the difference was not statistically significant (Figure 5—figure supplement 1). Thus, we conclude that EF3 plays a similar role across the K_V_7 family of CRDs.

## Discussion

As its name suggests, CaM is a CALcium MODULated protein, regarded as a fundamental player in the orchestration of Ca^2+^ signals in every eukaryotic cell (Clapham, 2007). Notwithstanding, its function is not limited to Ca^2+^ signaling, having important functions in protein trafficking to the plasma membrane, protein folding, and other functions (Villarroel et al., 2014). Here, the portfolio of CaM capacities is extended by providing evidence of its essential role in transducing redox signaling in conjunction with K_V_7 channels, which exhibit an exquisite sensitivity to oxidation. Current augmentation can be induced by an external H_2_O_2_ concentration as low as 5 μM or nM concentrations when using a faradaic device for delivery (Abdullaeva et al., 2022; Gamper et al., 2006), which is within the range of extracellular peroxide release due to dopamine oxidation in rat brain (Kulagina and Michael, 2003). Such high sensitivity places K_V_7 channels among candidate proteins that are first to respond to H_2_O_2_ production and reveals a prominent role of CaM in redox signaling.

Previously, we demonstrated the significance of cysteine residues in neuronal K_V_7 channels located in the unusually long (compared to other voltage-operated K^+^ channels) intracellular linker joining transmembrane segments S2 and S3, which are part of the voltage sensor (Gamper et al., 2006). Here, we show that the ability of the EF3-hand of CaM to bind Ca^2+^ is essential in this redox-signaling pathway. This is inferred, among others, from the observation that the effect of bath application of H_2_O_2_ is lost in cells overexpressing CaM variants with a disabled EF3-hand or treated with the membrane permeable Ca^2+^ chelator BAPTA-AM.

Earlier, we showed that the redox response depends on the presence of cysteine residues at the S2S3 loop (Gamper et al., 2006); yet, the number of cysteine residues is not the sole factor defining the efficacy of the response. No evidence for redox regulation was observed for WT K_V_7.1 channels (that have only one cysteine residue at the S2S3 loop); and engineered ‘three Cys K_V_7.1 channel’ displayed a weak response to H_2_O_2_. WT K_V_7.4 (three Cys residues in the S2S3) displayed a strong response to H_2_O_2_, yet a partial response was still observed for engineered ‘one Cys K_V_7.4 channels’ (Gamper et al., 2006). Hence, even with only one cysteine present, the S2S3 linker can mediate H_2_O_2_ sensitivity of a ‘responsive’ K_V_7 channel (such as K_V_7.4), while there must be other structural constrains that hinder potentiation of K_V_7.1 by the H_2_O_2_.

We suggest that a low channel open probability (p_o_) is an additional requirement for redox sensitivity because no evidence for redox regulation has been observed for K_V_7.3 channels, which present a p_o_ close to the unity at saturating voltages (Li et al., 2004). These channels have a triple Cys residue motive at the S2S3 loop that only differs at one position when compared to redox-sensitive K_V_7.2 channels (an Arg residue in K_V_7.3 versus a Lys residue in K_V_7.2). We suggest that due to very little room for further increase in p_o_, oxidation has negligible consequences on macroscopic K_V_7.3 currents.

The redox response is characterized by a remarkable increase/recovery in M-current density on a second to minute time scale, and it can be reversed by reducing agents (Abdullaeva et al., 2022; Gamper et al., 2006). This could derive from insertion of new channels, engaging silent channels, higher open probability, or a combination of these. Although the insertion/recruitment of new channels cannot be completely discarded, H_2_O_2_ causes an increase in single-channel activity in excised patches where the incorporation of new channels cannot take place. The redox impact on K_V_7 channels is accompanied by a left shift in the current-voltage relationship of macroscopic currents, meaning that channel opening becomes easier at lower voltages or that the probability of opening at a given voltage increases (Gamper et al., 2006). Large leftward shifts in voltage dependency of K_V_7 channels after over-expression of CaM C-lobe mutants (Chang et al., 2018; Sihn et al., 2016) have been reported, suggesting a critical role for EF3 (Chang et al., 2018). However, this shift has not always been observed (Archer et al., 2019). In this study, we saw relatively small (~10 mV) but consistent leftward shift in voltage dependence of K_V_7.4 co-expressed with all CaM mutants containing the EF3 mutation, suggesting a degree of tonic channel inhibition conferred via EF3.

Taking into account the images at atomic resolution of CaM interacting with the voltage sensor of K_V_7.1 channels (Sun and MacKinnon, 2017), it is tempting to propose that through this interaction, CaM is dragging the voltage sensor, making it more difficult to reach the up position that leads to gate opening in response to depolarizations, or CaM is stabilizing the state where the voltage sensor is disengaged from the pore. In other words, we envisage CaM constitutively and dynamically inhibiting channel activation and that the redox action relieves this inhibition by weakening the dynamic CaM-S2S3 interaction in a Ca^2+^-dependent manner. This idea fits with the observation that EF3 is not interacting with S2S3 in the available atomic resolution structures trapped in a partially (Sun and MacKinnon, 2020) and fully (Zheng et al., 2021) open states. To harmonize with other observations, we propose that this inhibition is counterbalanced by CaM-dependent promotion of surface expression when CaM or CaM1234 are over-expressed (Etxeberria et al., 2008; Gomis-Perez et al., 2017). Furthermore, our data suggest that Ca^2+^ binding to EF3 should help releasing the voltage sensor from CaM. In principle, this should lead to current potentiation, but the effect of Ca^2+^ on gating may be conditioned by other circumstances, as discussed next.

To what extend the observations can be extrapolated to the native channel is a concern raised when using peptides. Our MD simulations reveal that the N-terminal helix present in the channel structure of the S2S3 domain disappears when the peptide is in solution. In contrast, the C-terminal helix is remarkably stable. Nevertheless, the map of interactions between EF3 and CaM highlights the loop connecting these two helices, which overlap with the residues found or assumed to make contacts in the K_V_7/CaM complexes (Sun and MacKinnon, 2017). However, conclusions derived from the use of isolated peptides may not translate linearly to the whole channel complex. Interestingly, FRET changes in the isolated recombinant CRD caused by S2S3 peptides depend on the concentration of Ca^2+^ are reversibly sensitive to H_2_O_2_ treatment and primarily governed by EF3. The left-shift observed on the emission spectrum of D-CaM, a property observed only after loading EF-hands with Ca^2+^ (Alaimo et al., 2013), is consistent with a functional interaction of the peptides with the EF-hands in the loops coordinating Ca^2+^. The dose-response relationship with D-CaM and our MD simulations illustrates that the interaction is weaker when the EF3-hand is loaded with this cation. It is very clear that the influence of S2S3 on the relative orientation of helices A and B is only manifested in the presence of Ca^2+^, which is also a necessary condition for functional effects of H_2_O_2_ on K_V_7.4 currents. This is remarkable because S2S3 interacts with apo-CaM causing a shift on the peak emission of D-CaM (i.e. presumably, through EF-hands). The lack of any impact on energy transfer on the CRD reveals that the interaction under low Ca^2+^ conditions does not result in a conformational change in the AB fork. The additive increase in the magnitude of D-CaM fluorescent emission and left-shift in peak emission is indicative of the concurrent interaction of Ca^2+^ and the S2S3 loop with EF3, something that becomes apparent when examining the K_V_7.1/CaM complex (Sun and MacKinnon, 2017).

Remarkably, we find that EF3 is essential within the isolated recombinant CRD to translate Ca^2+^ signaling into conformational changes, which result in an 18° opening of the AB fork (Bernardo-Seisdedos et al., 2018). Thus, signaling through EF3 is a property inherent to the CRD and does not derive from constrains that the geometry of the voltage sensor-pore imposes. We note that the architecture of K_V_7 channels allows exploiting EF3 signaling in a more efficient way. We call attention on the conditional duality of this signaling system. One branch operates on the voltage sensor (S2S3/EF3), and another branch changes the orientation of helix A, likely affecting S6, and therefore, the main gate formed by the S6 bundle crossing. Whereas we propose that the S2S3/EF3 branch inhibits the current by disengaging the voltage sensor, the consequences of the relative movements of helix A are unclear. Helix A and S6 are connected by a conserved flexible linker whose helical character varies upon PIP_2_ binding, causing a variable S6-hA orientation relative to the gate (Li et al., 2021a; Li et al., 2021b; Niu et al., 2020; Sun and MacKinnon, 2020; Zheng et al., 2021). The conditional S2S3-dependent reorientation of helix A caused by Ca^2+^ is posited to favor or hinder pore opening depending on the initial orientation of the CRD, which, in turn, depends on PIP_2_ occupancy (Li et al., 2021b; Niu et al., 2020; Zheng et al., 2021). This complexity of interactions could explain contrasting effects of Ca^2+^ elevation. Whereas in rat superior cervical neurons (Selyanko and Brown, 1996) and CHO cells (Gamper and Shapiro, 2003; Kosenko and Hoshi, 2013), this cation inhibits M-currents, a clear current potentiation was observed in *Xenopus* oocytes (Gómez-Posada et al., 2011). Further experiments are required to clarify this issue. We note that our data are compatible with the proposed role of S2S3 and EF3 loops in Ca^2+^ K_V_7.4 regulation (Zhuang and Yan, 2020) and highlight an intricate conditional network of signaling processes, in which PIP_2_ binding, redox regulation, and Ca^2+^ are interconnected (Gomis-Perez et al., 2017).

An unexpected observation was the reversal in FRET index observed at higher S2S3 peptide concentrations only in the presence of Ca^2+^. Although our data suggest that S2S3 interacts with CaM in the presence and absence of Ca^2+^, occupation EF3 by this cation is a requirement to signal the reorganization of the CRD and for the functional redox effect on the channel. FRET changes caused by Ca^2+^ are opposed in the presence of S2S3 peptides. At the molecular level, we do not know what the reversal of the signal implies because any modification in distance or orientation causes FRET alterations. It seems reasonable to propose that, in this scenario, the movement within the AB helices goes in the opposite direction, leading to a tighter packing of the fork, which is consistent with increased FRET signal. Tighter packing is what is observed when comparing the K_V_7.2 fork in absence of S2S3 - with and without Ca^2+^- with the S2S3 loop interacting with EF3, presumably loaded with Ca^2+^ (Sun and MacKinnon, 2017; Figure 5B).

Given the structural similarities between the CaM lobes, it is remarkable that both the CSPs maps and the MD contact maps report mainly signals from the C-lobe. In reference to the MD data, it should be borne in mind that the initial condition corresponds to S2S3 interacting with EF3, which introduces a bias in favor of capturing interactions with the C-lobe. Regarding the NMR data, two observations provide clues that help explaining this apparent selectivity. On one hand, the displacements are, in general, of smaller magnitude in the presence of Ca^2+^. Incidentally, this goes along with the observation that the relative fluorescence signal from D-CaM with S2S3 and Ca^2+^ is about half of what is observed when Ca^2+^ is chelated. On the other hand, under basal conditions, the N-lobe sites are already occupied by this cation (Bernardo-Seisdedos et al., 2018), and therefore, the displacements are expected to be smaller to begin with. Nevertheless, even under high Ca^2+^ conditions (i.e. with the four EF-hands occupied), the overall displacements are observed preferentially at the C-lobe, consistent with an intrinsic preference for this lobe. Regarding the displacements observed at the N-lobe, a similar magnitude under the two experimental conditions explored could be expected since Ca^2+^ occupies the two sites at the N-lobe in both situations. However, there are some differences, especially at the initial portion of the N-terminus and at the EF2 binding loop. In addition, there is a notable difference between the MD contact map and the NMR CSPs within the residues connecting EF3 and EF4. We speculate that these differences are a consequence of allosteric EF-hand-coupling (Sorensen and Shea, 1998).

Based on docking calculations, the functional existence of significant interactions between a target protein and apo-CaM through the EF-hands of the C-lobe was first proposed for the smoothelin-like 1 protein (Ishida et al., 2008). Subsequently, direct interactions between apo-EF3 and myosin were observed in X-ray structures, and this interaction was postulated to play an important role in signal transduction (Münnich et al., 2014). Recent analysis of surface interaction from cryo-EM structures of ion channels with CaM hints to interactions between EF3 and EF4 with Eag1, TRPV5, and TRPV6 channels (Núñez et al., 2020). Thus, Ca^2+^ signal bi-directional transduction through direct interaction between the Ca^2+^-binding sites and CaM target protein could be a widespread mechanism.

## Materials and methods

**Key resources table:** 

Reagent type (species) or resource	Designation	Source or reference	Identifiers	Additional information
Peptide, recombinant protein	K_V_7.1 S2S3	Proteogenix		RLWSAGCRSKYVGVWGRLRFARKP
Peptide, recombinant protein	K_V_7.2 S2S3	Proteogenix		RIWAAGCCCRYRGWRGRLKFARKP
Peptide, recombinant protein	K_V_7.3 S2S3	Proteogenix		RIWAAGCCCRYRKGWRLFKFARKP
Peptide, recombinant protein	K_V_7.4 S2S3	Proteogenix		RVWSAGCCCRYRGWQGRFRFARKP
Peptide, recombinant protein	K_V_7.5 S2S3	Proteogenix		RIWSAGCCCRYRGWQGRLRFARKP
Recombinant DNA reagent	K_V_7.1 mtfp-hAB-Venus (residues I247-D456) in pPROEX HTc	This paper	NM_000218.2	Plasmid, Fluorescence sensor
Recombinant DNA reagent	K_V_7.2 mtfp-hAB-Venus (residues I310-D549), in pPROEX HTc vector	This paper	NM_172107.3	Plasmid, Fluorescence sensor
Recombinant DNA reagent	K_V_7.3 mtfp-hAB-Venus (residues I349-D556) in pPROEX HTc	This paper	NM_004519.3	Plasmid, Fluorescence sensor
Recombinant DNA reagent	K_V_7.4 mtfp-hAB-Venus (residues I315-D539) in pPROEX HTc	This paper	NC_060925.1	Plasmid, Fluorescence sensor
Recombinant DNA reagent	K_V_7.5 mtfp-hAB-Venus (residues I308-D527) in pPROEX HTc	This paper	NC_060930.1	Plasmid, Fluorescence sensor
Recombinant DNA reagent	CaM in pOKD4	Recombinant Human CALM2 in pOKD4 vector, GenScript	Genbank, NP_001292553.1	Plasmid, Calmodulin, human CALM2
Recombinant DNA reagent	hKCNQ4-eYFPc	Gamper et al., 2006	AF105202	Plasmid,KCNQ4 bound to YFP
Recombinant DNA reagent	hKCNQ4CCC/AAA -eYFPc	Mutant AF105202; Gamper et al., 2006		Plasmid, Mutant KCNQ4 bound to YFP
Recombinant DNA reagent	CaM 3 in pOKD4	GenScript	Genbank, NP_001292553.2	Plasmid, human CALM2, D93A mutation
Recombinant DNA reagent	CaM124 in pOKD4	GenScript	Genbank, NP_001292553.3	Plasmid, mutant human CALM2, D20A/D56A/D129A
Recombinant DNA reagent	CaM123 in pOKD4	GenScript	Genbank, NP_001292553.4	Plasmid, mutant human CALM2, D20A/D56A/D93A
Recombinant DNA reagent	CaM12 in pOKD4	GenScript	Genbank, NP_001292553.5	Plasmid, mutant human CALM2, D20A/D56A
Recombinant DNA reagent	CaM34 in pOKD4	GenScript	Genbank, NP_001292553.6	Plasmid, mutant human CALM2, D93A/D129A
Recombinant DNA reagent	CaM1234 in pOKD4	GenScript	Genbank, NP_001292553.7	Plasmid, mutant human CALM2, D20A/D56A/D93A/D129A
Recombinant DNA reagent	CaM WT in pCDNA3	GenScript	Genbank, NP_001292553.8	Plasmid, Calmodulin, human CALM2
Recombinant DNA reagent	CaM3 in pCDNA3	GenScript	Genbank, NP_001292553.9	Plasmid, human CALM2, D93A mutations
Recombinant DNA reagent	CaM34 in pCDNA3	GenScript	Genbank, NP_001292553.10	Plasmid, human CALM2, D93A/D129A mutations
Recombinant DNA reagent	CaM12 in pCDNA3	GenScript	Genbank, NP_001292553.11	Plasmid, human CALM2, D20A/D56A mutations
Recombinant DNA reagent	CaM124 in pCDNA3	GenScript	Genbank, NP_001292553.12	Plasmid, human CALM2, D20A/D56A/D129A mutations
Recombinant DNA reagent	CaM1234 in pCDNA3.1	GenScript	Genbank, NP_001292553.13	Plasmid, human CALM2, D20A/D56A/D93A/D129A mutations
Recombinant DNA reagent	K_V_7.4 in pCDNA3.1	GenScript	Genbank, NP_001292553.14	Plasmid, human K_V_7.4 channel
Recombinant DNA reagent	K_V_7.4CCC/AAA in pCDNA3.1	Gamper et al., 2006	Genbank, NP_001292553.15	Plasmid, human K_V_7.4 channel, C156A, C157A, C158V
Cell line (*Homo sapiens*)	Kidney (normal epithelial, embryo)	ATCC	HEK293	

Chemical compound and drug	5-(Dimethylamino)naphthalene-1-sulfonyl chloride, DNSCl	SIGMA-ALDRICH	CAS Number: 605-65-2	Dansyl chloride
Chemical compound and drug	Pierce DTT (ditiotreitol)	Thermo Scientific	CAT# 20290	DTT
Chemical compound and drug	Hydrogen peroxide solution	SIGMA-ALDRICH	CAS Number = 7722-84-1 / Pubchem ID = 57654227	H2O2 30% (w/w) in H2O, contains stabilizer
Chemical compound and drug	X2254	SIGMA-ALDRICH		XE-991
Chemical compound and drug	ab145545	Abcam		Retigabine
Chemical compound and drug	E2311	Promega		Fugene
Software and algorithm	PyMOL	The PyMOL Molecular Graphics System, Version 1.3 Schrödinger, LLC.		Use for molecular dynamics and figure preparation
Software and algorithm	Patchmaster V2	HEKA Instruments		
Software and algorithm	VMD	Humphrey et al., 1996		Use for molecular dynamics and figure preparation
Software and algorithm	NAMD	Phillips et al., 2020		Use for molecular dynamics

### Cell culture and transfection

Human embryonic kidney line 293 (HEK293T) cells from ATCC authenticated via STR profiling by Eurofins Genomics (ISO 17025) and confirmed negative for mycoplasma via PCR (EZ-PCR Mycoplasma detection kit, Generon, 20-700-20) were cultured to 80% confluence before passaging and used for experimentation between P10 and P40. Cells were grown in Dulbecco’s modified Eagle’s medium (DMEM Gibco) containing penicillin (100 U/mL; Sigma), streptomycin (100 µg/mL; Sigma), and 10% fetal calf serum (Sigma). HEK293 cells were cultured in 24 well plates for 24 hr prior to transfection and transfected with 300 ng of each plasmid using FuGene transfection reagent (Promega), according to the manufacturer’s instructions. Plasmids: hKCNQ4-eYFPc (AF105202) or hKCNQ4CCC/AAA -eYFPc a triple cysteine to alanine mutation in the S2–S3 linker (C156A, C157A, and C158A; Gamper et al., 2006) were co-transfected with WT CaM or lobe mutants. For BAPTA-AM experiments, cells were incubated with 10 µM cell-permeable BAPTA-AM (B1205 ThermoFisher) for 30 min prior to recordings. Cells were also bathed in 10 µM BAPTA-AM for the duration of the recording.

### Electrophysiology

Amphotericin B perforated configuration of patch clamp technique was used to record K_V_7 current using a HEKA EPC10 amplifier and Patchmaster V2 (HEKA instruments). Voltage dependence of activation was investigated using a standard IV protocol consisting of a train of square voltage pulses (1000 ms) from –80 mV to +20 mV in 10 mV increments with a 600 ms deactivating pulse back to –80 mV; pulses were applied with 2 s interval. In this protocol, the tail current amplitude elicited by the deactivating step was measured to remove the impact of driving force on the recording. Pipettes were pulled using a horizontal puller (Sutter P-97) and fire polished to typically 2–4 MΩ resistance. Upon entering whole-cell configuration, cell capacitance was nulled, and series resistance was typically below 10 MΩ.

Intracellular solution contained: 0.4 mg/mL Amphotericin B, 160 mM KCl, 5 mM MgCl2, 5 mM HEPES; pH adjusted to 7.4 using NaOH (all from Sigma).

Extracellular solution contained: 160 mM NaCl, 2.5 mM KCl, 1 mM MgCl_2_, 10 mM HEPES, 2 mM CaCl_2_, and 10 mM Glucose; pH adjusted to 7.4 with NaOH (all from Sigma).

### Expression and purification of the CaM/mTFP-K_V_7.2-hAB-Venus complex and CaM-Venus

The K_V_7.2-hAB segment (residues 316–532), K_V_7.1-hAB (residues 250–412), K_V_7.3-hAB (residues 349–556), K_V_7.4-hAB (residues 316–571), and K_V_7.5-hAB (residues 309–524) in complex with CaM and CaM-Venus recombinant protein were purified as previously described (Bernardo-Seisdedos et al., 2018).

### Mutant CaM

CaM3, CaM4, CaM123, CaM124, and CaM1234 in pOKD4 vector were synthesized by GenScript Biotech Corporation (Netherlands). CaM3 has mutation in the EF3 (D95A), CaM4 in the EF4 (D131A), CaM123 has mutations in EF1, EF2, and EF3 (D22A, D58A, and D95A), CaM124 has three alanine substitutions in the first, second, and fourth EF hands (D22A, D58A, and D131A), while CaM 1234 has substituted all EF hands (D22A, D58A, D95A, and D131A).

### Expression and purification of CaM

Recombinant CaM was produced in BL21 DE3 bacteria and purified as previously described (Yus-Najera et al., 2002).

### Peptide design and solubilization

Proteogenix prepared and purified by reverse phase HPLC (High performance liquid chromatography) chromatography all peptides, with greater than 95% purity (Supplementary file 1) and stored at –20°C lyophilized. Peptides were solubilized first in DMSO to get 10 mM concentration. To obtain 100 µM K_V_7.2-S2/S3 solution, they were diluted in Fluorescence Buffer (Hepes 50  mM [pH 7.4], KCl 120  mM, NaCl 5  mM, MgCl_2_ 2  mM, and EGTA 5  mM) with 1 mM DTT.

### Reversible peptide oxidation by H_2_O_2_

A 30% solution of H_2_O_2_ was from Thermo Fisher Scientific. The DMSO-peptide stock at 10 mM was diluted in oxidizing buffer (120 mM KCl, Hepes 50 mM pH 7.4, 5 mM EGTA, 5 mM NaCl, and 1 mM H_2_O_2_ [0.003%]). The oxidized peptide was added to samples with 500 nM FRET-sensor, both KCNQ2 and KCNQ4, in the absence and in the presence of 1 mM CaCl_2_.

To reverse the oxidation, DTT was added at 1 mM to the previously oxidized peptide stock and incubated at 4°C O/N. Samples were prepared exactly the same, and FRET was analyzed as previously described.

### FRET experiments

All FRET experiments were carried using an AMINCON Bowman series 2 luminescence fluorimeter. The fluorescence emission spectra of the proteins at 500 nM were collected over 470–570 nM after excitation at 458 nm (2 nm bandwidth). The total protein in each condition was assessed by direct excitation of the yellow protein at 520 nm, collecting the emission at 520–570 nm. FRET index was established as the ratio of emission at 520–525 divided by emission at 485–490 nm upon excitation at 456–460 nm.

### Fluorometric measurements using D-CaM

Fluorescent D-CaM (5-[dimethylamino]naphtalene-1-sulfonyl-calmodulin) was prepared using recombinant CaM and dansyl chloride. Prior to the experiments, D-CaM and other proteins were dialyzed for 48 hr against 2 L of fluorescence buffer containing Hepes 50  mM (pH 7.4), KCl 120 mM, NaCl 5 mM, MgCl_2_ 2 mM, and EGTA 5 mM, changing the buffer every 12 hr. Steady-state fluorescence measurements were obtained at 25°C on a SLM SPF-8100 (Olis, inc) fluorescence spectrophotometer in a final volume of 100 μL. The fluorescence was measured after exciting at 340 nm and recording emission from 450 to 600  nm. Slit widths were set at 2 nm for excitation and 2 nm for emission.

Titration experiments were performed by adding increasing concentrations S2/S3 peptides to D-CaM (50 nM) in fluorescence buffer. Experiments were also performed in the presence of an excess of Ca^2+^ by adding 4.34 mM Ca^2+^ to the fluorescence buffer. The free Ca^2+^ concentration was determined using Fura-2 (Invitrogen), following the manufacturer’s instructions.

Fluorescence enhancement was plotted against the peptide concentration to generate the concentration-response curves. The parameters of the Hill equation were fitted to the data by curvilinear regression, enabling the apparent affinity (EC_50_ or concentration that gives half-maximal change in the intensity of the fluorescence emission) or the t_50_ (half-time or time that gives half-maximal change in the intensity of fluorescence emission). The data are shown as the average of three or more independent experiments.

### NMR measurements

Uniformly ^15^N-labeled K_V_7.2hAB:CaM was prepared in M9 medium containing 1 g/L ^15^NH_4_Cl as source of nitrogen. Other steps involved in the expression and purification of ^15^N-labeled K_V_7-2hAB:CaM were the same as for mTFP-K_V_7.2hAB-Venus:CaM. The ^2^D-^1^H,^15^N-HSQC experiments were performed by dissolving 75 µM complex in a buffer consisting of 50 mM Tris-HCl, 100 mM KCl, 10% D_2_O, and 10 mM EGTA (apo-Complex) or, alternatively, 5 mM CaCl_2_ (holo-Complex). The pH values of the sample solutions were carefully adjusted to 7.4 with trace amount of 2 M KOH. All NMR experiments were carried out at 25°C on a Bruker Avance III 600 MHz NMRs spectrometer. The HSQC (Heteronuclear single quantum correlation) spectra were acquired with a spectral width of 30 ppm in the ^15^N dimension and 16 ppm in the ^1^H dimension. In CSP experiments, peptide-CaM complexes were prepared by adding 13 equivalents of the K_V_7.1-S2S3 lyophilized peptide directly to the Q2-*h*AB-CaM NMR sample. The CSP studies were performed by monitoring the changes in the ^1^H,^15^N HSQC spectra of ^15^N-labeled CaM. The CSP values were then evaluated as a weighted average chemical shift difference of ^1^H and ^15^N resonances, using the equation:

.$$\delta =\sqrt{\left(0.1*\delta_{N}\right)2^{}+\;\delta_{H^{2}}}$$

### MDs simulations

Two systems were set up: the intermediate- (Int-CaM, N-lobe loaded with Ca^2+^) and the holo-CaM/K_V_7.2 hAB complexes (both lobes loaded with Ca^2+^) in the presence of the K_V_7.1 S2S3 peptide (164-RLWSAGCRSKYVGVWGRLRFARKP-187; PDB ID: 5VMS; Sun and MacKinnon, 2017). CaM/hAB complexes were modeled starting from the NMR structures of the K_V_7.2hAB/CaM complex (PDB IDs: 6FEG and 6FEH, for int- and holo-complexes, respectively; Bernardo-Seisdedos et al., 2018). The initial location of the peptide was established after aligning the EF3 lobes of the K_V_7.1/CaM and K_V_7.2/CaM complexes. Hydrogen atoms were added to the complexes using the VMD software (Humphrey et al., 1996). Two or four Ca^2+^ atoms were added to the int- and holo-systems, located as in PDB 6FEG and 6FEH, respectively. The systems were then solvated resulting in a cubic box of dimensions described in Supplementary file 2. K^+^ and Cl^+^ ions were added using the Autoionize plugin of VMD to neutralize the system resulting in a final concentration of 150 mM KCl. The S2S3 peptide was also considered alone in solution to investigate its structural stability. Several replicas were run for some of the systems (Supplementary file 2).

Simulations were performed with NAMD2.13 (Phillips et al., 2005). The CHARMM36 force field (Klauda et al., 2010) was used to model the protein and ions, and the TIP3P model was chosen for the water. NBFIX corrections for Ca^2+^ ions were adopted (Yoo et al., 2016). The Particle Mesh Ewald method was used for the treatment of electrostatic interactions, with an upper threshold of 1 Å for grid spacing (Darden and Pedersen, 1993). Electrostatic and van der Waals forces were calculated in every time step with a 12 Å cut-off distance. A switching distance of 10 Å was chosen to smoothly truncate the non-bonded interactions. Only atoms in a Verlet pair list with a 13.5 Å cut-off distance (reassigned every 20 steps) were considered. The SETTLE algorithm was used to constrain all bonds involving hydrogen atoms to allow the use of a 2 fs time step. The Nose-Hoover-Langevin piston method was employed to control the pressure with a 50 fs period, 25 fs damping constant, and a desired value of 1 atmosphere. The system was coupled to a Langevin thermostat to sustain a temperature of 298°K throughout.

The VMD software was used for analysis of the trajectories and production of some of the figures. All replicas for each system were merged, and contacts of the Cα of all the residues were computed in the analysis of the contacts between S2S3 and CaM. The contacts were then normalized to the total time and to the value of the residue with maximum contacts.

## Data Availability

All data generated or analysed during this study are included in the manuscript and supporting file.

## Additional files

MDAR checklist

Supplementary file 1. S2S3 petides information.

Supplementary file 2. Details of the molecular dynamics simulations.
