# Supplementary figures and images for: Redox regulation of KV7 channels through EF3 hand of calmodulin
Source: eLife. 2023 Feb 20;12:e81961. doi: 10.7554/eLife.81961 (PMC9988260; doi:10.7554/eLife.81961)

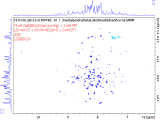

Supplement: Figure 4—source data 2. [file elife-81961-fig4-data2.zip › 20/pdata/1/thumb.png]

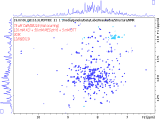

Supplement: Figure 4—source data 2. [file elife-81961-fig4-data2.zip › 21/pdata/1/thumb.png]

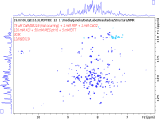

Supplement: Figure 4—source data 2. [file elife-81961-fig4-data2.zip › 22/pdata/1/thumb.png]

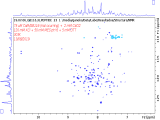

Supplement: Figure 4—source data 2. [file elife-81961-fig4-data2.zip › 23/pdata/1/thumb.png]
