# Supplementary material for: Redox regulation of KV7 channels through EF3 hand of calmodulin
Source: eLife. 2023 Feb 20;12:e81961. doi: 10.7554/eLife.81961 (PMC9988260; doi:10.7554/eLife.81961)
Supplement: Supplementary file 1. [file elife-81961-supp1.docx]

**Supplementary Table 1.** **S2S3 peptides information.**

Hydrophobicity of each peptide was estimated by Peptide 2.0 Inc (https://www.peptide2.com/N_peptide_hydrophobicity_hydrophilicity.php).

| **ISOFORM** | **SEQUENCE** | **PURITY** | **HIDROPHOBICITY** |
| --- | --- | --- | --- |
| K_V_7.1 | Ac-RLWSAGCRSKYVGLWGRLRFARKP-NH_2_ | >95% | 41.7% |
| K_V_7.2 | Ac-RIWAAGCCCRYRGWRGRLKFARKP-NH_2_ | >95% | 37.5% |
| K_V_7.3 | Ac-RIWAAGCCCRYKGWRGRLKFARKP-NH_2_ | >95% | 37.5% |
| K_V_7.4 | Ac-RVWSAGCCCRYRGWQGRFRFARKP-NH_2_ | >95% | 33.3% |
| K_V_7.5 | Ac-RIWSAGCCCRYRGWQGRLRFARKP-NH_2_ | >95% | 33.3% |
