# Supplementary material for: Redox regulation of KV7 channels through EF3 hand of calmodulin
Source: eLife. 2023 Feb 20;12:e81961. doi: 10.7554/eLife.81961 (PMC9988260; doi:10.7554/eLife.81961)
Supplement: Supplementary file 2. [file elife-81961-supp2.docx]

**Supplementary Table 2.** Details of the molecular dynamics simulations conducted in this study.
